# Dynamic behaviour restructuring mediates dopamine-dependent credit assignment

**DOI:** 10.1038/s41586-023-06941-5

**Published:** 2023-12-13

**Authors:** Jonathan C. Y. Tang, Vitor Paixao, Filipe Carvalho, Artur Silva, Andreas Klaus, Joaquim Alves da Silva, Rui M. Costa

**Affiliations:** 1 Department of Neuroscience, Zuckerman Mind Brain Behavior Institute, Columbia University, New York, NY USA; 2 Seattle Children’s Research Institute, Center for Integrative Brain Research, Seattle, WA USA; 3 Department of Pediatrics, University of Washington School of Medicine, Seattle, WA USA; 4 Champalimaud Neuroscience Programme, Champalimaud Research, Champalimaud Foundation, Lisbon, Portugal; 5 Kinetikos, Coimbra, Portugal; 6 Open Ephys Production Site, Lisbon, Portugal; 7 Champalimaud Experimental Clinical Research Programme, Champalimaud Research, Champalimaud Foundation, Lisbon, Portugal; 8 NOVA Medical School, Universidade NOVA de Lisboa, Lisbon, Portugal; 9 Aligning Science Across Parkinson’s Collaborative Research Network, Chevy Chase, MD USA; 10 Allen Institute, Seattle, WA USA

**Keywords:** Operant learning, Reward

## Abstract

Animals exhibit a diverse behavioural repertoire when exploring new environments and can learn which actions or action sequences produce positive outcomes. Dopamine release after encountering a reward is critical for reinforcing reward-producing actions^1–3^. However, it has been challenging to understand how credit is assigned to the exact action that produced the dopamine release during continuous behaviour. Here we investigated this problem in mice using a self-stimulation paradigm in which specific spontaneous movements triggered optogenetic stimulation of dopaminergic neurons. Dopamine self-stimulation rapidly and dynamically changes the structure of the entire behavioural repertoire. Initial stimulations reinforced not only the stimulation-producing target action, but also actions similar to the target action and actions that occurred a few seconds before stimulation. Repeated pairings led to a gradual refinement of the behavioural repertoire to home in on the target action. Reinforcement of action sequences revealed further temporal dependencies of refinement. Action pairs spontaneously separated by long time intervals promoted a stepwise credit assignment, with early refinement of actions most proximal to stimulation and subsequent refinement of more distal actions. Thus, a retrospective reinforcement mechanism promotes not only reinforcement, but also gradual refinement of the entire behavioural repertoire to assign credit to specific actions and action sequences that lead to dopamine release.

## Abstract (web summary)

Initial dopamine self-stimulations reinforced not only the stimulation-producing target action, but also actions similar to the target action and actions that occurred a few seconds before stimulation, and repeated pairings led to a gradual refinement of the behavioural repertoire to home in on the target actions.

## Main

Animals spontaneously transition among a repertoire of movements when exploring new environments. Movements or movement sequences that produce positive outcomes are reinforced and increase their frequency to maximize the production of such outcomes^4,5^. However, it is not completely clear how animals assign credit to the exact action that produces a reward in the context of a continuous behavioural space. This credit-assignment problem^2,6–9^ during spontaneous behaviour poses at least two main challenges. First, it is unclear how animals refine their behaviour to preferentially perform a specific reward-producing action compared to similar actions in the behavioural repertoire. Second, it is unclear how animals derive contingency between reward-producing actions or action sequences and reward if there can be variable delays between action or sequence performance and reward delivery, with many actions interleaved.

Dopamine (DA) has been proposed to mediate credit assignment^6,10^. At the cellular level, DA can facilitate synaptic plasticity in corticostriatal synapse^11^ within a critical time window that is behaviourally relevant^12–14^. Still, it is unclear how DA changes the dynamics of spontaneous behaviour to mediate credit assignment. We therefore developed a paradigm to investigate how DA shapes the evolution of continuous behaviour during action learning to gain insights into the process of credit assignment.

**Fig. 1:**
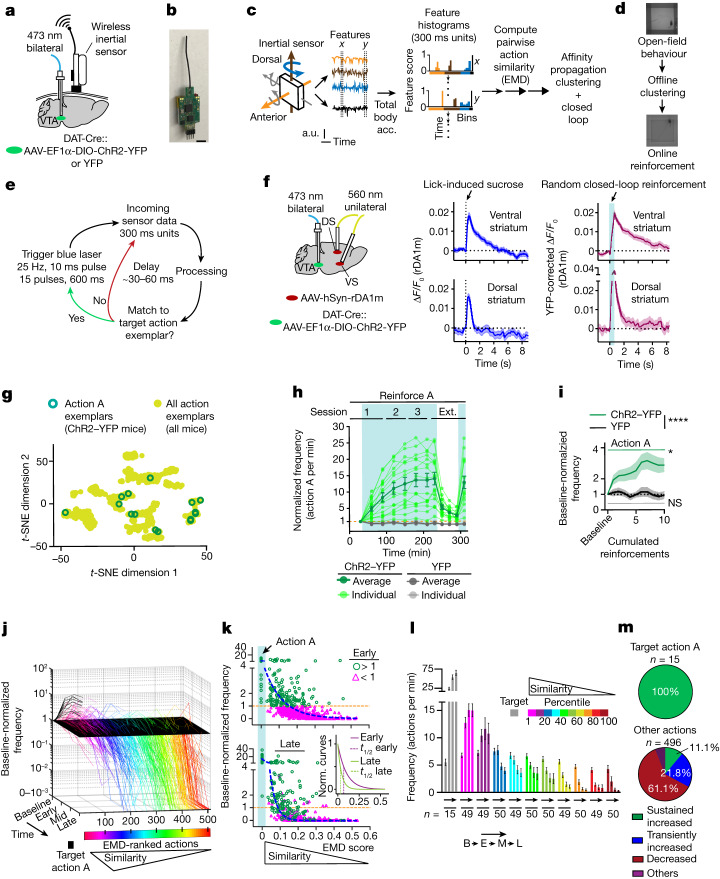
Learning of a single action from the naive state as mediated by closed-loop optogenetics. **a**, Animal implant schematic. **b**, Wireless inertial sensor. Scale bar, 0.5 cm. **c**, Sensor data processing. Acc., acceleration; a.u., arbitrary units. **d**, Open-field behavioural clustering and action reinforcement. **e**, Closed-loop schematic. **f**, DA release in the dorsal (DS) and ventral (VS) striatum. *n* = 70 sucrose rewards, *n* = 2 ChR2–YFP mice (biological replicates); *n* = 66 and *n* = 65 random stimulations, *n* = 2 ChR2–YFP and *n* = 2 YFP mice (biological replicates), respectively. Data are mean ± s.e.m. **g**, Action A exemplar locations in behavioural space. **h**–**m**, ChR2-dependent reinforcement of action A. *n* = 15 (ChR2–YFP; green) and *n* = 10 (YFP; grey) mice. Data are mean ± s.e.m. **h**, Frequency changes in action A. The light green and grey lines represent individual ChR2–YFP and YFP mice, respectively. Ext., extinction. **i**, Rapid increase in target action performance in response to closed-loop reinforcements. Statistical analysis was performed using repeated-measures two-way analysis of variance (ANOVA); significant time × group interactions are indicated (*F*_35,805_ = 3.12; *P* = 7.7 × 10^−9^). Data are mean ± s.e.m. **j**, Evolution of the pooled behaviour repertoire (*n* = 509 actions, ChR2–YFP mice) across learning. **k**, Early/late cross-sectional views of **j** (early: baseline normalized (norm.) frequency of >1 (green circles) and <1 (magenta triangles)). The blue dashed lines show single phase log decay fits. Inset: early/late fitted lines normalized to 1 at EMD = 0. **l**, Raw frequencies across learning and target similarity percentile groups. *n* values indicate the sample size (actions). Data are mean ± s.e.m. Two-way mixed-effects statistics are provided in the Supplementary Information. B, baseline; E, early; L, late; M, mid. **m**, The distribution of actions according to their dynamics within reinforced action A (left) or other actions (right). ****P < 0.0001, *P < 0.05; NS, not significant. Statistical and sample details are provided in the Supplementary Information. Source Data

Conventional operant conditioning paradigms^5,15–19^ have helped to extract principles of reinforcement. However, they usually require animals to interact with devices (for example, levers, nosepokes, joysticks) or perform a series of consummatory actions to obtain reward, all of this in specific locations in space. These aspects make it difficult to investigate how credit is assigned to a specific action or action sequence of the entire repertoire during continuous behaviour. We developed an approach to study credit assignment whereby we directly reinforce the execution of specific spontaneous movements by triggering DA neuron excitation and DA release after their execution, irrespective of where in space they are executed. The approach combines wireless inertial sensors, unsupervised clustering of continuous behaviour^20,21^ and optogenetics^22^ in a closed-loop system linking specific action performance to immediate phasic DA release (Fig. 1a–e and Methods). This paradigm reinforces actions without requiring an animal to approach or interact with a place, object or cue, or to perform consummatory behaviour.

## Rapid closed-loop reinforcement of actions

**Extended Data Fig. 1:**
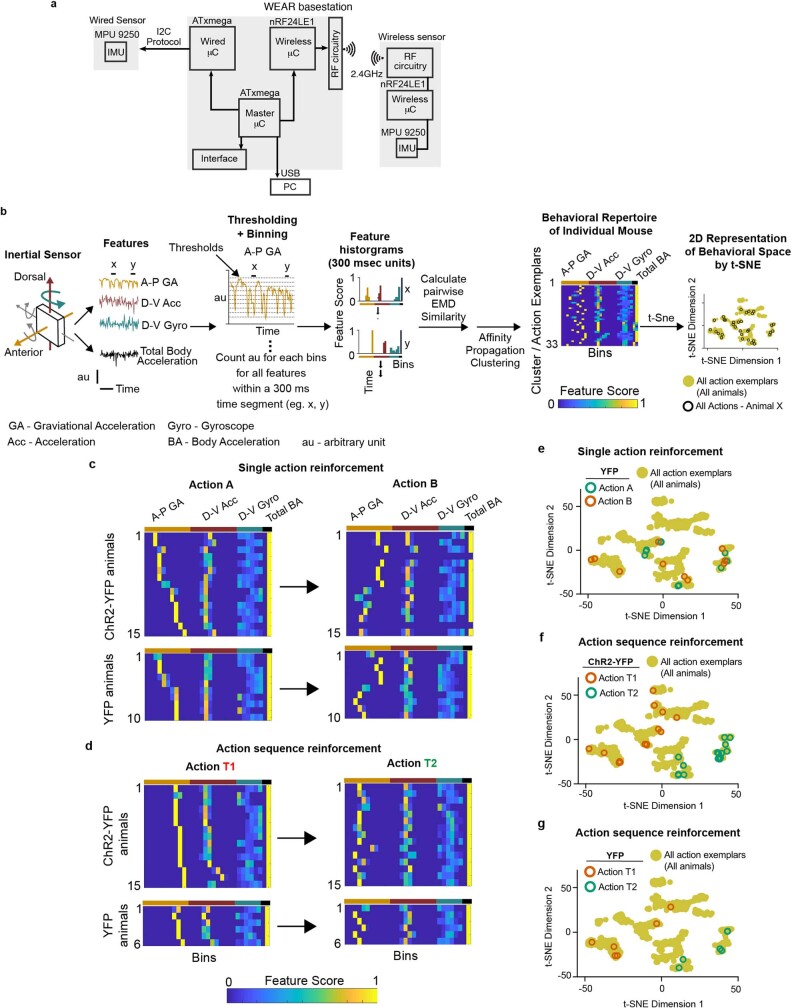
Behavioural monitoring hardware, action clustering and selected actions for reinforcement. **a**, Schematic showing the WEAR basestation and communications with either wired (not used in this study) or wireless inertial sensors. **b**, Detailed schematic showing processing of inertial sensor data for behavioural clustering and 2-D behavioural repertoire visualization. **c**, Heat map showing motion feature score distributions of exemplar Action A (left) and Action B (right) reinforced for each animal. **d**, Heat map showing motion feature score distributions of exemplar Action T1 (left) and Action T2 (right) reinforced for each animal. **e-g**, t-SNE plots showing 2-D behavioural space showing all action exemplars pooled from all animals (yellow circles) overlaid with actions targeted in single action reinforcement YFP (e), two action ChR2-YFP (f) and YFP (g) animals. Source Data

**Extended Data Fig. 2:**
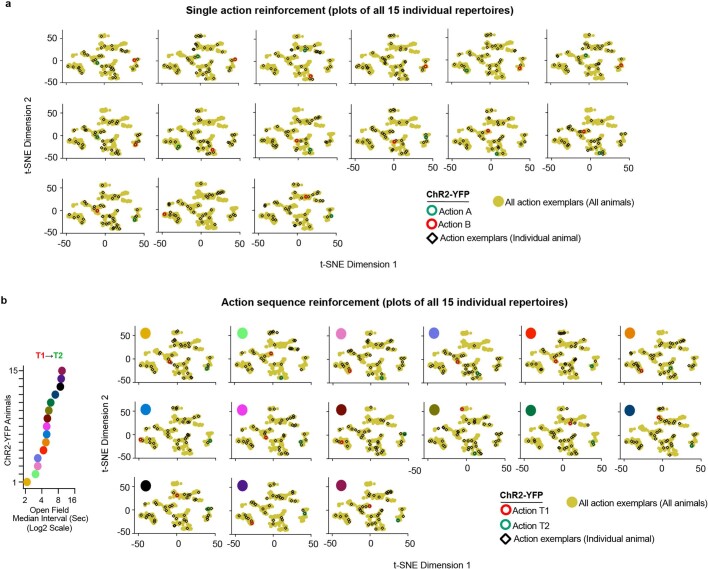
Behavioural spaces of individual ChR2-YFP animals used in this study. **a-b**, t-SNE plots showing distribution of all action exemplars in individual ChR2-YFP animals across the 2-D behavioural space (yellow circles, pooled from all animals) in single action (**a**) and action sequence (**b**) learning. **b**, The plots of action sequence learners are ordered according to their initial open field median T1→T2 intervals. Leftmost graph: Open field inter-action intervals of T1/T2 pairs chosen. Same colour codes in right t-SNE graphs. *n* = 15 ChR2-YFP animals (biological replicates) in each experiment. Source Data

To implement closed-loop reinforcement, we used a Cre-dependent strategy to express channelrhodopsin ChR2–YFP^22^ or control YFP bilaterally in DA neurons of the ventral tegmental area (VTA)^23,24^ of DAT-Cre mice^25^ (Fig. 1a, Methods Extended Data Fig. 2a,b). We classified the entire behavioural repertoire of individual mice in a grey-walled open field (Fig. 1b–d). Self-paced behaviour was monitored using a wireless inertial sensor system (WEAR; Methods) that enables minimal movement restraints, high-resolution behaviour monitoring and fast data transmission to open-source hardware and software for online experimentation (Fig. 1b and Extended Data Fig. 1a). Affinity propagation clustering was applied to classify behaviour, as it is advantageous for identifying an unknown number of clusters^20,21^, is computationally efficient^26^ and easily outputs similarity between clusters (Methods and Supplementary Methods). We identified over 30 clusters of spontaneous behaviour per individual (34.3 ± 2.1 and 35.6 ± 2.5 actions per ChR2–YFP and YFP mice, respectively (mean ± s.d.); 15 ChR2–YFP and 10 YFP mice), representing the entire behaviour repertoire of each individual in the open field. For each animal, we chose to reinforce two clusters, or actions (hereafter, actions A and B), that are highly mobile and tend to have opposing feature score distributions in the anterior–posterior postural and dorsal–ventral head/body turn features (Methods and Supplementary Video 1). The sampled action pairs are quite dissimilar as measured by Earth-mover’s distance (EMD)^27^, enabling us to assess the learning process for two distinct action types per animal. Variation in features across actions was allowed to extract generalizable principles common to action learning.

Although movement transitions are also accompanied by DA release in the substantia nigra pars compacta^28,29^ and this release can pattern behaviour^30^, VTA DA release after encountering unexcepted rewards is pronounced and strongly reinforces behaviours that lead to it^23,24,31,32^. We therefore targeted VTA DA neurons and stimulated them with a pattern (25 hz, 600 ms long train^33^) that would mimic DA release after encountering sucrose, rather than substantia nigra pars compacta DA neurons (Supplementary Methods). Target actions were different between animals and dispersed across a behavioural space (Fig. 1g and Extended Data Figs. 1 and 2). To evaluate whether stimulation parameters triggered DA release similar in magnitude to that triggered by a sucrose reward in food-restricted mice, we monitored DA release under both conditions with the GRAB rDA1m sensor^34^ in both the ventral and dorsal striatum (Fig. 1f). Sucrose presentation led to a sharp increase in DA release in both areas (Fig. 1f). Notably, random optogenetic stimulation of DA neurons in the VTA with the parameters described above resulted in a similar phasic increase in DA not only in the ventral striatum but also in the dorsal striatum, although there is a relatively higher DA release in the dorsal striatum (Fig. 1f). This is consistent with evidence of dorsal-striatum-projecting VTA neurons^29,35^. Thus, our optogenetic stimulation triggered DA release similar in decay and spatial localization to that triggered by sucrose rewards in food-restricted mice (Fig. 1f), offering a suitable approach to examine how pairing DA release with the performance of specific actions leads to credit assignment.

Over a 3 day, 60–90 min per session protocol designed to probe both intra- and intersession changes in behaviour, closed-loop stimulation of VTA DA neurons after execution of a particular target action (action A) significantly increased target action frequency for ChR2–YFP, but not YFP mice (Fig. 1h and Extended Data Fig. 4a,b). The increased frequency of action A depends on optogenetic stimulation, as demonstrated by both extinction and reinstatement of stimulations (Fig. 1h and Extended Data Fig. 4c,d). During extinction, ChR2–YFP mice kept performing exploratory unrewarded bursts of action A, which could explain the rapid reinstatement (Extended Data Fig. 4e,f). Just a few pairings with DA leads to rapid reinforcement, as changes in multiple parameters, including decreased interval between triggers, increased action A frequency and increased average behavioural similarity towards action A, become significant after 10–15 stimulations (Fig. 1i, Methods and Extended Data Fig. 5a,b).

We next examined the effect of closed-loop reinforcement on non-stimulated actions. We tracked the baseline-normalized frequency of all actions while sorting them on the basis of similarity to the target action, using EMD^21,27^ (Fig. 1j). A lower EMD value indicates increased similarity. Notably, optogenetic stimulation substantially changed the entire behavioural repertoire performed in the open field. Early on, actions most similar to the target action tended to also increase in frequency (Fig. 1j–l and Extended Data Fig. 5c), whereas actions most dissimilar to the target action tended to decrease in frequency. Repeated pairing led to refinement of actions that were performed at a high frequency and, by late stages, action A became the predominant action being performed, with a sharp decrease in non-target-action frequencies as the similarity to the target action decreased (Fig. 1k,l). Such effects were not observed in the YFP control mice (Extended Data Fig. 5d–f). Thus, early reinforcement results in rapid reshaping of the entire behavioural repertoire, biasing animals towards actions similar to the target action, and continued pairing results in gradual refinement and assignment of credit to the specific target action.

## Dynamic behavioural refinement with reinforcement

To better describe individual action dynamics during reinforcement, the trajectories of all action frequency changes throughout learning were categorized (Methods; 511 actions, *n* = 15 ChR2–YFP animals). Three meaningful types of trajectories were characterized by ‘sustained increase’, ‘transient increase’ and ‘decreased’ dynamics (94% of all actions; Fig. 1m, Supplementary Methods and Extended Data Figs. 6 and 7). The dynamics of action reinforcement were, yet again, related to the action’s similarity to the target action, regardless of whether the actions were sorted on the basis of their raw or percentile similarity scores (Extended Data Fig. 7b,c). Actions most similar to the target action were predominately sustained-increase types. Moderately similar actions mostly comprised of sustained-increase or transient-increase types. Highly dissimilar actions tend to be decreased types. Transiently increased dynamics of similar actions were not caused by stimulation of non-target actions, as misclassification of the target for stimulation was very rare (1.02 × 10^−5^ mismatches per actual triggers, 1 in 97,924 triggers, 15 ChR2–YFP animals) and target distributions do not overlap with most other actions in the two-dimensional *t*-distributed stochastic neighbour embedding (*t-*SNE) behavioural space (Extended Data Fig. 8a,b). Importantly, these transiently increased clusters show little to no overlap with target distribution^36^ and did not lead to DA triggers (Supplementary Notes and Extended Data Fig. 8c). Thus, target misclassification was not a major cause of transiently increased action dynamics. These results show that the dynamics of action reinforcement are greatly related to the similarity to the target action, in that animals initially increase re-entrance of similar actions before homing in on the target action.

## Learning new action-reward contingencies

**Fig. 2:**
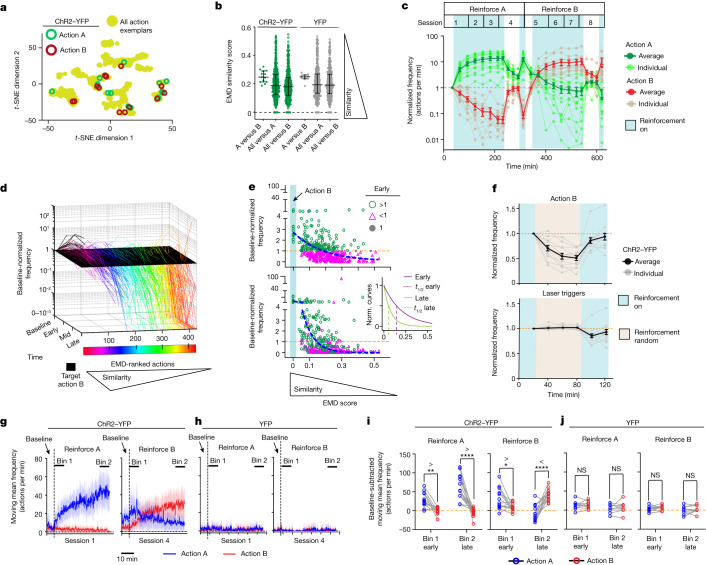
Transitioning from a learned action to reinforcing a new action. **a**–**j**, Animals (biological replicates) reinforcing for action A (*n* = 15 ChR2–YFP) to action B (*n* = 13 of 15 ChR2–YFP). *n* = 10 (YFP). *n* values indicate animals (biological replicates). **a**, Action A and B exemplar locations in behavioural space. **b**, Action similarity comparisons (A versus B: *n* = 13 (ChR2–YFP) and *n* = 10 (YFP); all versus A: *n* = 514 (ChR2–YFP) and *n* = 356 (YFP); or all versus B: *n* = 443 (ChR2–YFP) and *n* = 356 (YFP)). Data are median ± interquartile range. **c**, Reinforcement for action A and B in ChR2–YFP mice. Data are mean ± s.e.m. **d**, Evolution of the pooled action repertoire (*n* = 427 ChR2–YFP actions) reinforced for action B. **e**, Early/late cross-sectional views of **d**. The blue dashed lines indicate the fitted decay curve. Inset: normalized early/late fitted curves. **f**, Contingency degradation of action B. The random laser trigger frequency (bottom) is based on the initial action B performance before contingency degradation. Data are mean ± s.e.m. **g**–**j**, Action A (blue) induced by reinforcement for action B in experienced ChR2–YFP (**g**,**i**) and YFP (**h**,**j**) mice. **g**,**h**, Moving-mean frequencies over reinforcement for action A or B. The dashed vertical lines mark the first reinforcement. Data are mean ± s.e.m. (coloured fill). Bin 1 and bin 2 are time bins for **i** and **j**. **i**,**j**, Frequency measures within the time bins noted in **g** and **h**. Statistical analysis using repeated-measures two-way ANOVA reveals a significant difference across time and action A/B frequencies (ChR2–YFP: reinforce A, *F*_1,24_ = 34.4, *P* = 4.8 × 10^−6^; reinforce B, *P* = 1.2 × 10^−8^); two-sided Šidák’s post hoc multiple comparisons test was applied. ****P < 0.0001, **P < 0.01, *P < 0.05; NS, not significant. Statistical and sample details are provided in the Supplementary Information. Source Data

We next examined whether animals could follow changes in contingency between action and closed-loop DA stimulation. We therefore chose a different action, action B, which is clearly distinct from the action A for each animal (Fig. 2a, Methods and Extended Data Fig. 1c) and started delivering DA stimulation after action B. Chosen action A–B pairs were relatively dissimilar in the context of entire action similarity distributions (Fig. 2b). After reinforcement, previously trained ChR2–YFP mice, but not YFP mice, showed increased action B performance over time and the action A frequency was reduced to the baseline levels (Fig. 2c–e and Extended Data Fig. 9). Maintenance of action B performance depended on continual reinforcement (Extended Data Fig. 9f,g). Similar to action A, action B credit assignment unfolds by initially biasing the entire repertoire towards performance of actions similar to target B and away from dissimilar actions. This was again followed by gradual refinement for action B relative to similar actions over pairings (Fig. 2d,e and Extended Data Fig. 9h). We confirmed that action learning is contingent on action B happening before reinforcement by degrading the contingency between action B and DA, by giving random stimulations in the same average frequency but unpaired to action B; the contingency was reinstated after resuming action B–stimulation pairings (Fig. 2f and Extended Data Fig. 9i). Although similar patterns of behavioural refinement were observed for actions A and B, differences in the initial responses were noted (Fig. 2g–j and Supplementary Notes). Still, animals can follow changes in the contingency between actions and DA release and assign credit to a new action through a similar process of behavioural repertoire refinement.

## Temporal constraints of DA-dependent reinforcement

Reinforcement is thought to occur on behaviour that precedes a reward in time^10,12,14,19^, and temporal contiguity between action and reinforcement has long been recognized^37–39^ and was observed above (Fig. 2f). We therefore investigated whether, in addition to behavioural similarity, the temporal relationship between action and stimulation influenced the dynamics of behavioural repertoire evolution during reinforcement and credit assignment.

**Fig. 3:**
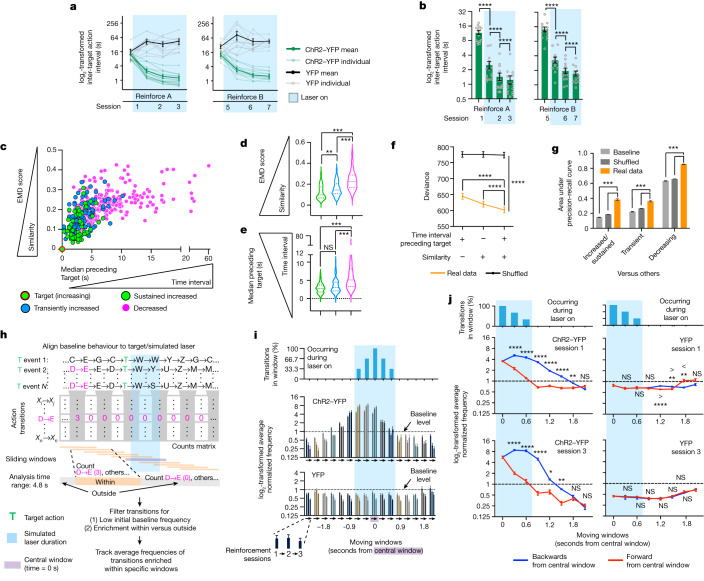
DA mediates retrospective reinforcement of freely moving behaviour. **a**,**b**, ChR2-dependent reinforcement decreases inter-action intervals for action A (**a**; *n* = 15 ChR2–YFP) and B (**b**; *n* = 13 of 15 ChR2–YFP). *n* = 10 (YFP). *n* values indicate animals (biological replicates). For **a** and **b**, data are mean ± s.e.m. Significant differences across time and ChR2–YFP/YFP are indicated (mixed-effects model: action A: *F*_3,69_ = 72.26, *P* = 3.0 × 10^−21^; action B: *F*_3,62_ = 33.78, *P* = 4.6 × 10^−13^). For **b**, post hoc two-tailed Tukey’s test was applied for multiple-comparison analysis of the data shown in **a**. **c**–**e**, The distribution of action dynamic types (*n* = 464 (non-target actions), 15 (target actions) and 15 (ChR2–YFP mice)) according to target similarity and median time to target (**c**), target similarity (**d**) and median time to target (**e**). For **d** and **e**, the violin plots show the median and quartiles. Statistical analysis was performed using two-tailed permutation tests; Bonferroni-adjusted *P* values are shown. **f**,**g**, Multinomial logistic regression of all factor combinations in real data (200 independent models) versus shuffled data (10,000 independent models, 50 independently shuffled datasets). Baseline, 200 independent models. **f**, The two-factor model fits data better than one-factor models. Groups differ across combinations (repeated-measures two-way ANOVA; *F*_2,30,594_ = 1,082, *P* = 0.0 × 10^0^). Two-tailed post hoc Dunnett multiple-comparisons test was applied. Data are mean ± s.d. **g**, The performance of the double-factor regression model was determined using the area under the precision-recall curve criterion. Statistical analysis was performed using two-tailed permutation tests; Bonferroni-adjusted *P* values are shown (*P* = 5.9 × 10^−4^, all comparisons). Data are mean ± s.e.m. **h**, The pipeline for identifying sliding-window-enriched action transitions. D→E, arbitrary action transition. **i**, ChR2-dependent reinforcement for action A increases sliding-window-enriched action transitions before and during stimulation. The average normalized frequency of action transitions enriched within specific sliding windows was plotted over sessions 1–3. Top, the percentage of transitions occurring during stimulation in each sliding window. Middle and bottom, the mean ± s.e.m. normalized frequency of action transitions. **j**, Quantification of **i**. Data are mean ± s.e.m. Significant differences across time and retrospective/forward reinforcement directions are indicated (mixed-effects modelling; ChR2–YFP session 1: *F*_6,168_ = 114.8, *P* = 8.7 × 10^−57^; ChR2–YFP session 3: *F*_6,168_ = 46.62, *P* = 2.5 × 10^−33^; YFP session 1: *F*_6,108_ = 10.52, *P* = 3.6 × 10^−9^; YFP session 3: *F*_6,168_ = 0.8992, *P* = 0.49). Post hoc two-sided Šidák multiple-comparison test was applied. ****P < 0.0001, ***P < 0.001, **P < 0.01, *P < 0.05; NS, not significant. Statistical and sample details are provided in the Supplementary Information. Source Data

The median inter-target action interval decreased with stimulation in ChR2–YFP mice (Fig. 3a,b and Methods). We therefore examined the distribution of the action dynamic types categorized above (Extended Data Fig. 7a) according to both (1) an action’s similarity to the target action and (2) the median time of that action’s performance leading to the target action during the baseline, before reinforcement (Fig. 3c–e). These two dependent variables were not significantly collinear (Methods). Multinomial logistic regression showed that action similarity and baseline temporal proximity to the target action together predict action dynamic type after reinforcement better than either factor alone (Fig. 3f,g, Methods, Supplementary Methods and Supplementary Table 1). Thus, DA reshapes the behavioural repertoire by reinforcing both actions similar to the target action and actions that happen to be performed temporally close to the reinforcer, as suggested previously^12^.

To more rigorously test whether DA reinforcement acts in a retrospective or prospective manner, we refined our analysis by examining first-order action transitions leading into and out of stimulation (Fig. 3h–j and Methods). Action transitions enriched within specific 1.2 s sliding windows were analysed to distinguish more clearly behaviour that occurred leading up to, during and after DA stimulation (Methods). We identified baseline-occurring action transitions enriched within specific sliding windows centred around the target action and tracked their average frequencies per window over the course of closed-loop reinforcement. Action transitions that were enriched in windows up to 1.2 s before stimulation onset, as well as during stimulation, were reinforced early on (Fig. 3i). However, action transitions that were enriched in windows after stimulation were not reinforced, suggesting an asymmetric process. Indeed, action transitions enriched in windows leading into stimulation were also preferentially reinforced over those enriched in windows after stimulation (Fig. 3j). Thus, DA stimulation promotes the reinforcement of behaviours occurring during stimulation and a few seconds before stimulation.

## Credit assignment for action sequences

**Fig. 4:**
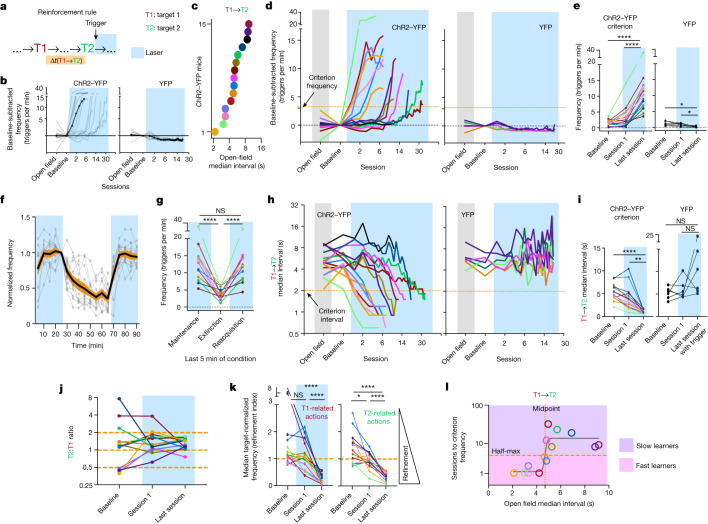
The relationship between pre-reinforcement inter-action intervals and learning of a two-action sequence. **a**, Experiment schematic. **b**, ChR2-dependent increase in T1→T2 triggers (no laser during open field/baseline). **c**, Open-field inter-action intervals of the T1/T2 pairs chosen. The same colour codes are used for ChR2-YFP mice in **d–e** and **g–l**. **d**, Individual learning curves labelled according to the colours in **c**. For **c**, **d** and **h**, a log_2_-scale *x* axis was used. **e**, Frequency changes over conditions. Statistical analysis was performed using repeated-measures one-way ANOVA (*F*_1.911,24.85_ = 51.02, *P* = 2.2 × 10^−7^). **f**,**g**, Extinction of T1→T2 sequence (ChR2–YFP). **f**, Data are mean (black line) ± s.e.m. (orange shading), and individuals (grey lines). **g**, Frequency changes over extinction conditions. Statistical analysis was performed using repeated-measures one-way ANOVA (*F*_1.073,12.87_ = 52.96, *P* = 9.8 × 10^−6^). **h**,**i**, ChR2-dependent decrease in T1→T2 intervals in ChR2–YFP (**h**) and YFP (**i**) mice. Statistical analysis was performed using repeated-measures one-way ANOVA (*F*_1.377,17.90_ = 35.95, *P* = 1.5 × 10^−5^) (**i**). The log_2_-scale *y* axis was used to help to visualize interval changes in animals starting with lower initial values. **j**, T2:T1 frequency ratios (ChR2–YFP). **k**, Target refinement shown by median target-action-normalized frequencies of related actions. Statistical analysis was performed using repeated-measures one-way ANOVA (T1: *F*_1.237,16.08_ = 43.38; T2: *F*_1.171,15.22_ = 48.74; both: *P* = 4.4 × 10^−6^). **l**, A sigmoidal relationship between the open-field T1→T2 interval and the number of sessions to the criterion frequency. The log_10_-scale *y* axis was used to capture the relationship between large values and smaller values in the same visualization. For **b**–**l**, *n* = 15 (**b**, **d** and **h**), 14 (**e** and **i**–**l**) or 13 (**f** and **g**) ChR2–YFP mice and 6 YFP mice (biological replicates). For **e**, **g**, **i** and **k**, statistical analysis was performed using repeated-measures one-way ANOVA with post hoc Šidák test. For **b** and **d–l**, plots of individual mice are shown. ****P < 0.0001, **P < 0.01, *P < 0.05; NS, not significant. Source Data

**Extended Data Fig. 3:**
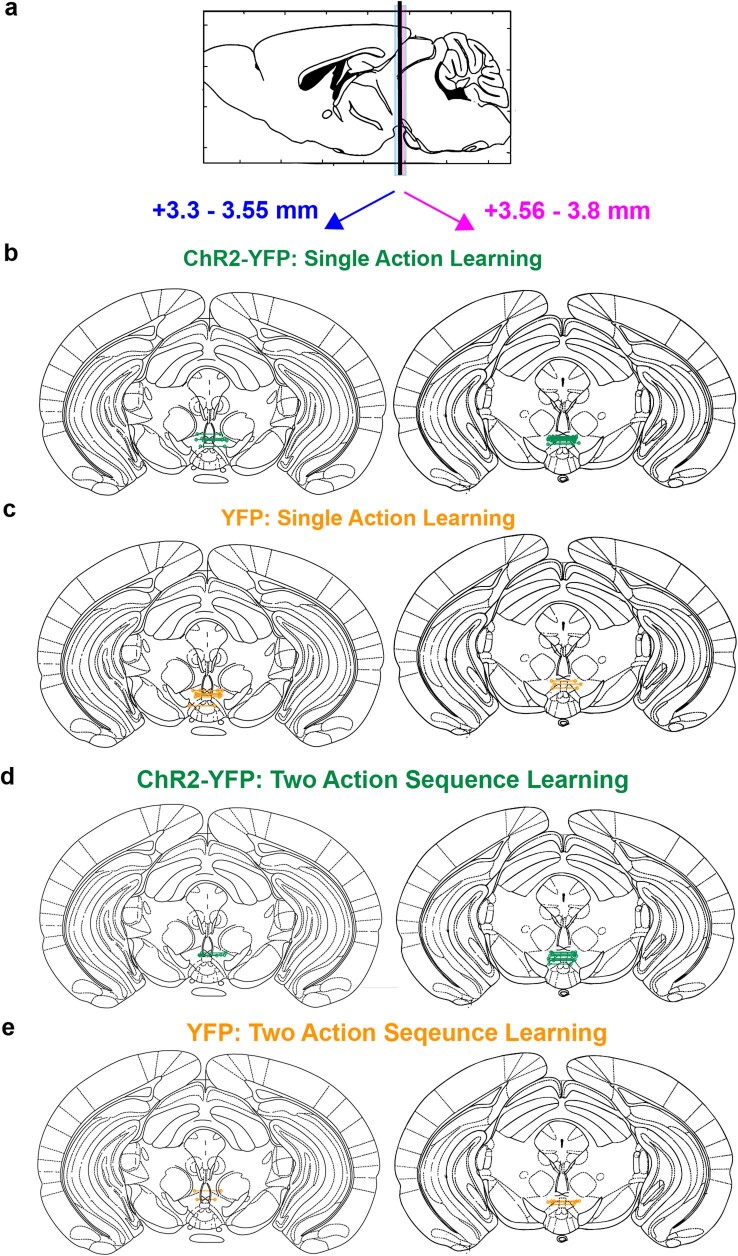
Implant locations for all animals used in this study. **a**, Sagittal view of mouse brain with labelling of anterior (blue) and posterior (magenta) brain regions surrounding injection/implant coordinates (vertical black line). **b-c**, Actual dual cannula implant locations of individual ChR2-YFP (*n* = 15; green) (**b**) and YFP (*n* = 10; orange) (**c**) animals used for single action reinforcement experiments. (n: animals - biological replicates) **d-e**, Actual dual cannula implant locations of individual ChR2-YFP (*n* = 15; green) (**d**) and YFP (*n* = 6; orange) (**e**) animals used for two action sequence reinforcement experiments. Individual dual implant locations are marked by open circles joined by horizontal lines. Source Data

In the real world, when animals are spontaneously shifting between actions in their repertoire, outcomes are often not the result of a single action but, rather, are the result of a sequence of actions performed at variable time intervals, and with other actions interleaved. We therefore investigated the dynamics of reinforcement when the release of DA was contingent on performance of a sequence of two target actions, target actions 1 (T1) and 2 (T2), whereby variations in the time interval between the two target actions, as well as interleaving actions were allowed. We applied closed-loop optogenetics to examine whether naive animals can learn a T1→T2 reinforcement rule, whereby the delays between T1 and T2 are governed by the spontaneous behaviour of the animals and are not experimentally controlled (*n* = 15 (ChR2–YFP) and *n* = 10 (YFP) mice; Fig. 4a and Extended Data Figs. 2b, 3a,d,e and 10–14). Various T1–T2 pairs were sampled, with a focus on sequences sharing general commonalities in movement order across animals (Methods and Extended Data Fig. 1d,f,g). Overall, the mice learned to increasingly perform these sequences to obtain DA stimulation. Some animals showed a ChR2-dependent increase in reinforcement within 5 sessions, but others experienced a lag in learning (Fig. 4b). We hypothesized that this relates to the initial time distance between T2 trigger and the closest distal T1 (T1→T2 interval). Indeed, animals reinforced for action pairs with initially long interval values tended to learn slower (Fig. 4c,d). To capture a timepoint at which individuals reach similar rising phase in their respective learning curves, a criterion frequency was set (Methods). In total, 14 out of 15 trained mice eventually reached the criterion (Fig. 4e and Extended Data Fig. 10). Sequence performance depended on continuing DA pairings (Fig. 4f,g). Learning was also revealed by decreases in the median T1→T2 time intervals (Fig. 4h,i) and convergence of the T1-to-T2 frequency ratio towards 1 (Fig. 4j). To quantify the specific credit assignment of T1 and T2, we used a refinement index that compares the median frequency of actions that are uniquely similar to T1 with those that are uniquely similar to T2, with the frequencies normalized to either that of T1 or T2 (Methods). This index is based on the observation that actions that are most similar to the target action decrease in relative performance over time (Fig. 1k (inset)). Values of less than 1 indicate greater refinement. By the end of learning, T1 and T2 became credited as the reward-producing actions relative to their similar counterparts (Fig. 4k). YFP controls did not show learning trends (Fig. 4d,e,h,i). Thus, closed-loop reinforcement promoted the learning of a two-action sequence rule in freely moving mice starting from a naive state.

Importantly, the initial median T1→T2 interval of action pairs was inversely related to the eventual number of sessions required for each ChR2–YFP animal to reach criterion frequency (Fig. 4l). A sigmoidal curve was fit to the data, indicating that initial intervals that were longer than the sigmoidal midpoint were associated with slower learning (Fig. 4l). ChR2–YFP animals were divided according to the half-maximum point of the sigmoidal curve into 'fast learners’ and ‘slow learners’. Fast learners quickly reached the criterion frequency and low T1→T2 time intervals, whereas slow learners were delayed in reaching the criterion frequency and low T1→T2 intervals. Slow learners tended to suddenly increase sequence frequency in sessions that showed a decrease in the median T1→T2 interval to below 2–4 s (Fig. 4d,h). By contrast, there was no stable sigmoidal relationship between T1–T2 action similarities and the number of sessions to the criterion frequency (Extended Data Fig. 11b). Furthermore, there was no relationship between the baseline frequency or initial inter-trigger intervals and the number of sessions to the criterion frequency (Extended Data Fig. 11c,d). Importantly, the observed patterns held when we analysed learning by matching the number of reinforcements (Extended Data Fig. 11e–g), indicating that they were not caused by fast learners having more stimulations/reinforcers. Lastly, we evaluated whether differential conditionability^40–43^ accounts for sequence learning differences (Extended Data Fig. 12). Target actions that showed less conditionability in single-action reinforcement did not differ in initial baseline frequencies but tended to have more action types transitioning into and out of them at the baseline (Extended Data Fig. 12d–f). Thus, differential conditionality among target actions relates to greater variation in the behavioural environment surrounding the target action. However, the same parameters do not account for variation in the learning rate across animals in the action sequence reinforcement experiment (Extended Data Fig. 12g–l). These results support the idea that the initial median time distances between distal action T1 and proximal action T2 (which produced DA stimulation) modulated how fast animals learned to effectively perform the reinforced action sequence.

**Fig. 5:**
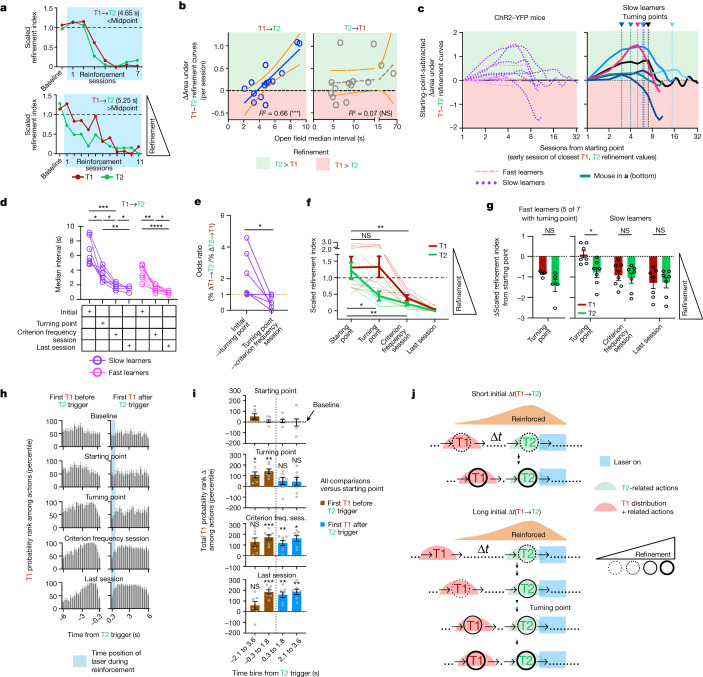
The behavioural process underlying the learning of a two-action sequence. **a**, T1/T2 refinements in two ChR2–YFP individuals. **b**, The linear relationship between the initial T1→T2 interval and differential T1–T2 refinement (*F* test, non-zero slope significance: T1→T2, *P* = 0.0004; T2→T1, *P* = 0.7063). **c**, Progression of differential T1–T2 refinement from the starting point in individual learners. **d**, The T1→T2 interval significantly decreased by the turning point in slow learners. Repeated-measures two-way ANOVA was used to analyse the time-specific difference (slow learners, *F*_2.184,26.20_ = 54.21, *P* = 5.3 × 10^−10^; fast learners, *F*_1.700,20.40_ = 92.12, *P* = 6.3 × 10^−9^). Post hoc two-tailed Tukey’s multiple-comparison test was applied. **e**, The odds ratio of T1→T2/T2→T1 interval changes in slow learners. Statistical analysis was performed using two-tailed paired Wilcoxon tests (*P* = 0.0312). **f**, Preferential refinement of T2 relative to T1 by the turning point in slow learners. Raw scaled refinement indices are shown. A repeated-measures mixed-effects model was used to analyse the significant main effects (time: *F*_2.184,26.20_ = 54.21, *P* < 0.0001). Post hoc two-sided Šidák multiple-comparison test was applied. **g**, Starting-point-subtracted scaled refinement indices. Left, fast learners. Statistical analysis was performed using a Welch’s two-tailed *t*-test. Right, slow learners. A repeated-measures mixed-effects model was used to analyse the difference between time and group (*F*_3,36_ = 4.276, *P* = 0.011). Post hoc two-tailed Šidák multiple-comparison test was applied. **h**, Ranking, among all actions, of the probability that the first T1 occurs within specified time bins before (left) and after (right) T2 triggers across learning. **i**, Quantification of pooled time bins from **h**. freq. sess., frequency session. Repeated-measures two-way ANOVA was used to analyse learning stage versus rank change. First occurrence of T1 before and after T2 trigger groups differ across learning stage and total T1 probability rank change (proximal bins (0.3–1.8 s): *F*_3,36_ = 3.126, *P* = 0.0376; distal bins (2.1 to 3.6 s): *F*_3,36_ = 7.701, *P* < 0.001). Post-hoc two-tailed Šidák multiple-comparison tests were applied for all learning stage values compared with starting-point values. **j**, Models for learning T1→T2 sequences differing in initial time separations. Time is not drawn to scale. Data are mean ± s.e.m. *n* = 14 ChR2–YFP (7 slow (**a–i**) and 7 fast (**c**,**d**,**g**) learners) mice (biological replicates). ****P < 0.0001, ***P < 0.001, **P < 0.01, *P < 0.05; NS, not significant. For **f**–**i**, statistical and sample details are provided in the Supplementary Information. Source Data

If DA retrospectively reinforces actions performed earlier in time, the action most proximal to reinforcement, T2, should experience earlier refinement relative to the distal action, T1. We again used the median target-normalized frequencies of actions uniquely related to T1 or T2 as refinement indices (Methods). T2 clearly refines towards its most refined level earlier than T1, at least in some of the animals (Fig. 5a). We calculated differential refinement between the two actions by subtracting the area under the T1 refinement curve from that of T2. Positive values indicate differential refinement favouring T2, and vice versa. The open-field median T1→T2 interval was linearly related to the differential refinement between T1 and T2 (Fig. 5b). This trend holds even when accounting for within-session refinement (Methods and Extended Data Fig. 13a). Thus, for longer T1→T2 median intervals, T2 spends more sessions being relatively more refined than T1, and this pattern cannot be explained by other potential covariates: (1) initial intervals between the proximal action and the next initiation of sequence (T2→T1); and (2) similarity between T1 and T2 (Fig. 5b (right) and Extended Data Fig. 13b).

The increased differential refinement favouring the proximal T2 could reflect increased refinement of T2 or reflect reduced refinement of distal T1 without refinement for T2. To distinguish between these interpretations, we analysed changes in T1–T2 refinement curves relative to the ‘starting points’ at which the refinement indices of T1 and T2 are most similar or are biased towards T1 rather than T2 (Methods). Slow learners initially showed differential refinement favouring T2 from these starting points and, after reaching a maximum differential refinement favouring T2 (called the turning point), refinement begins to turn towards favouring T1 (Fig. 5c). By the turning points, the median intervals of T1→T2, but not T2→T1, events decreased significantly relative to the initial values (Fig. 5d and Extended Data Fig. 13c). Thus, the median T1→T2 interval decrease occurred before a decrease in the interval to perform the next sequence (T2→T1) (Fig. 5e). Using these learning landmarks, we investigated more rigorously how animals homed in on T1 versus T2 over time (Fig. 5f,g). Animals initially refined the action proximal to stimulation (T2), whereas T1 refinement occurred later, after the turning point (Fig. 5f,g). By contrast, fast learners show relatively little differential refinement over learning (Fig. 5c and Extended Data Fig. 14b,c). Finer temporal analyses revealed that T1 was increasingly likely within the seconds preceding T2 reinforcement events by the turning point (Fig. 5h,i and Methods), even though T1 refinement was not yet apparent (Fig. 5f). After the turning point, T1 refinement and increased sequence performance coincide with T1 becoming significantly more probable within seconds after T2 reinforcement events (Fig. 5h,i), indicating increased sequence reinitiation. These results demonstrate how animals can assign credit to sequences of temporally distant target actions that lead to reinforcement, following retrospective dynamics predicted by single-action credit assignment. Specifically, actions that are most proximal to reinforcement are refined early on and the actions that are more distal to reinforcement become refined later, when they probabilistically start to occur within a few seconds of DA release.

## Discussion

Our results show that DA promotes credit assignment to specific actions and action sequences from a naive state through a dynamic process whereby the entire behavioural repertoire is restructured and refined. During initial reinforcements, there is a rapid increase in the frequency of not only the target action, but also of actions in the repertoire that are similar to the target action. However, dissimilar actions decrease in frequency. This rapid restructuring of the entire behavioural repertoire based on similarity to the target action facilitates the credit-assignment process. There is also an increase in actions that occur within a precise time window of a few seconds before and during, but not after, VTA DA neuron stimulation. With repeated reinforcement, gradual refinement unfolds to home in on the action that produces DA release. In the case of action sequences, both target actions in the sequence gradually become credited relative to their most similar actions. However, there is an interaction between the dynamics of refinement of the different target actions in the sequence and the temporal proximity to DA release. When sequences naturally varying in temporal separations between the two targets were reinforced, sequences with a naturally short temporal distance between the two targets tend to refine together. However, credit assignment for sequences with naturally long temporal distances between the two targets is accomplished by earlier refinement for the actions that are most temporally proximal to reinforcement, followed by later refinement for the more temporally distal actions.

Previous synaptic and cellular studies^12,14^ proposed that DA reinforcement may act retrospectively to reinforce behaviour. Using the closed-loop system, we rigorously tested this prediction. As retrospective reinforcement of behaviour is not confined to the target action alone, it facilitates credit assignment to a stimulation-producing action, even when reinforcement is delayed; stimulation-producing action pairs that tend to be performed close together in time were learned much faster than pairs that tended to be performed far apart in time. Notably, animals eventually learned to assign credit to distal stimulation-producing actions even in the latter scenario. This is characterized by a gradual process whereby, early on, the median time interval between distal and proximal target actions decreased and the repertoire proximal to reinforcement was preferentially refined. As the distal target became significantly more likely to occur within a distance at a timescale of seconds before reinforcement, retrospective reinforcement of the correct stimulation-producing sequences became increasingly likely, resulting in whole behavioural refinement for the distal target as well, therefore increasing sequence performance (Fig. 5j). This study has caveats that should be mentioned. The behavioural repertoire of the mouse in the open field is limited compared with more complex, naturalistic conditions. Furthermore, although our stimulation parameters produced similar DA release to that triggered by unsignalled sucrose consumption, these are not identical conditions. Thus, the complexity of behavioural repertoire, and the frequency, duration and exact placement of DA activation^42^ could affect the exact window and rate of refinement/reinforcement. However, the revealed principles of reinforcement and refinement during credit assignment should be generalizable.

Retrospective reinforcement of behaviour^4,19^ is thought to be mediated by DA modulation of an eligibility trace left by action-potential-triggered synaptic plasticity^10^. Studies of DA action at the striatal synaptic level^12,14^ indicate that retrospective reinforcement may occur on the order of a few seconds, but the behavioural consequences have remained unclear until now. Our behavioural findings agree with cellular studies that behaviour occurring within a few seconds leading into DA stimulation are reinforced. Furthermore, our findings reveal an interaction between refinement process and temporal proximity to DA release—refinement becomes more pronounced if target actions occur within a few seconds before stimulation. The cut-off of retrospective reinforcement and refinement by phasic DA activities could explain the increase in sessions required to reach the criterion frequency among animals that were reinforced for action pairs with initially longer median time separations. Similar actions have more similar and overlapping striatal neural ensemble activities^21^. The arrival of DA after the activation of action-specific ensembles may reinforce not only a specific action, but also similar actions. As striatal ensembles specific to actions are activated and a trial of eligibility traces is left temporally, DA arrival could mediate retrospective reinforcement of a spatially graded repertoire of actions within a few seconds, resulting in the observed behavioural learning patterns. Future studies would clarify how synaptic plasticity and cellular ensemble activities integrate to produce a dynamic refinement process, resulting in the behavioural principles for credit assignment revealed here.

## Methods

### Animals

All of the experiments were approved by the Portuguese DGAV and Champalimaud Centre for the Unknown Ethical Committee and performed in accordance with European guidelines. They were also performed according to National Institutes of Health (NIH) guidelines and approved by the Institutional Animal Care and Use Committee of Columbia University. DAT-Cre male mice (*Mus musculus*; aged 3–5 months) in the C57/BL6J background (JAX, 006660)^25^ were used. Animals were heterozygous for the transgene and generated from a cross between heterozygote DAT-Cre male mice with C57/BL6J female mice. The mice were housed under standard light cycles (08:00–20:00 light, 20:00–08:00 dark).

### Sample sizes, randomization and blinding

For sample size, we applied a power of 0.8, significance of *P* < 0.05 and standard variation of 20% of the mean. We determined sample sizes of 4–8 mice per group for different mean-based tests (matched pairs, two groups). No formal method of randomization was used; littermates were equally divided among the groups being compared. The experimenter was not blinded to the experimental groups. Optogenetic manipulations were performed automatically by a computer algorithm and not manually by the experimenter.

### Recombinant adeno-associated viral vectors, stereotaxic injections and implants

A total of 750 nl of rAAV.EF1a.DIO.hChR2(H134R).eYFP or rAAV.EF1a.DIO.eYFP (3–4 × 10^12^ viral genomes per ml, AAV5, University of North Carolina Vector Core; 1–2 × 10^13^ viral genomes per ml, AAV1, Addgene, 27056-AAV1 and 20298-AAV1) were injected into each hemisphere of the VTA of 3–4-month-old DAT-Cre mice. For viral injections, the coordinates were as follows: AP, +3.52 mm; ML, ±0.35 mm; DV, 4.3 mm. Injections were made at 0.2 Hz pulses. Each pulse injects 4.6 nl volume. Injected needles were kept in place in the injection site for around 15 min before withdrawal. For each mouse, a dual-optic fibre cannula (200/240 μm diameter, 6 mm length, 0.7 mm centre-to-centre FLT, 0.22 NA; Doric, DFC_200/240-0.22_6mm_DF0.7_FLT) was placed 200 μm above the injection site and fixed to the skull. Next, a four-position receptacle connector (Harwin, M52-5000445) was fixed anteriorly to the dual-optic fibre cannula, with its posterior edge set at +0.6 mm. Skull implants were then fixed with dental cement. A four-position connector (Harwin, M52-040023V0445) with pins removed from one end was used to cap the receptacle connector.

For photometry experiments, 3–5-month-old DAT-Cre male mice were used. The conditions used for VTA injections and implants were as described above. Moreover, 1 μl and 500 nl of AAV9-hSyn-GRAB-rDA1m (2 × 10^13^ viral genomes per ml; Addgene, 140556-AAV9) were injected into the dorsal striatum (AP, –0.5 mm; ML  +2.1 mm (right), DV, 2.3 mm (from the brain surface)) and ventral striatum (AP, –1.15 mm; ML, +1.65 mm (right); DV, 4.2, from bregma)), respectively. For photometry fibre implants, mono fibreoptic cannula were used (400/430 μm diameter, 4 mm length (dorsal striatum) and 6 mm length (ventral striatum), 0.37 NA, 1.25 diameter ferrule, flat; Doric, MFC_400/430-0.37_6mm_MF1.25_FLT (ventral striatum) and MFC_400/430-0.37_4mm_MF1.25_FLT (dorsalstriatum)). Implants were inserted at a 22° angle. For the dorsal striatum implantation, the cannula entered the skull at AP –0.5 mm and ML +3.03 mm at a 22° angle. The angled implant penetrated the brain from its surface for 1.92 mm. For ventral striatum implantation, the cannula entered the skull at AP –2.85 mm at a 22° angle, ML +1.65 mm. The angled implant penetrated the brain from its surface for 4.25 mm. Stereotaxic injections and implants information deposited on protocols.io (10.17504/protocols.io.8epv5xdw4g1b/v1).

### WEAR motion sensor system

The WEAR motion sensor family was developed by the Champalimaud Hardware platform and Costa laboratory as a wired or wireless solution to obtain self-centred nine-axis motion data based on three-axis accelerometer, gyroscope, and magnetometer (https://www.cf-hw.org/harp/wear). The wired version is a very small and extremely lightweight device (200 mg) that can sample motion data at up to 500 Hz and, at the same time, provide current up to 500 mA that can be used to power LEDs for optogenetic experiments or stimulating electrodes. The wireless version is small and lightweight (~1.8 g) and can sample motion data up to 200 Hz while having the ability to provide up to 50 mA that can be used to power LEDs for optogenetic experiments or stimulating electrodes. The battery of the wireless WEAR enables recordings of up to 4 h at 200 Hz sampling rate and even longer at lower sampling rates. These devices communicate with the computers through a base station based on the HARP design developed by the Champalimaud Hardware Platform, which can be accessed through a software GUI to easily change sensor parameters to best fit the experimental needs. The base stations have several important hardware features such as two digital inputs and outputs, an analogue input, two outputs for camera triggering and a clock sync input and output that provides hardware-based synchronization. The sensor can be started or stopped by software or pin. The WEAR motion sensor family and base station are all open source (repository at https://bitbucket.org/fchampalimaud/workspace/projects/HP). Moreover, the WEAR devices are compatible with the Bonsai visual reactive programming software (https://bonsai-rx.org/), also open source, and enable the integration and synchronization of the streams of data being collected using the WEAR sensor with other data sources such as cameras.

Taking these specs and features together, the WEAR device enables researchers to acquire high-resolution motion data wirelessly and for long periods of time without being computationally very demanding. The nine-dimensional motion data acquired through WEAR are simple to process, easy to connect to analysis software, enabling the fast online behaviour classification that was fundamental for the experiments described in this Article.

### Open-field experiment

One-month after surgery, mice were habituated to head-mounted equipment over 2 days. On day 1, an actual or mock wireless inertial sensor (~2.5 cm height × 1 cm length × 0.5 cm width, with ~2.5–3.0 cm antennae, around 1.8 g weight) glued to the four-position connector (Harwin, M52-040023V0445) was attached to the implanted receptacle connector on the skull cap. Individual mice roamed freely in the home cage for 1 h. On day 2, an actual wireless inertial sensor and mono fibreoptic patchcord (200/220 μm diameter, 0.22 NA; Doric DFP_200/220/900-0.22_2m_DF0.7-2FC) was attached to the skull cap through a mating sleeve. Patchcords were attached to a 1x2 fibre-optic rotary joint (intensity division, 0.22 NA; Doric, FRJ_1x2i_FC-2FC) and mice roamed freely in the home cage for 1 h. On the open-field recording day, the sensor/patchcord habituated mice were anaesthetized by isoflurane, attached to equipment, processed for the calibration protocol described below and individually placed into an open-field box inside a sound-insulated chamber. The open-field box is made of 410 × 400 mm grey opaque acrylic walls and a 410 × 400 mm white matte acrylic base. Individual mice were allowed to behave freely inside the box for 75 min. The wireless inertial sensor (~1.8 g in weight, WEAR wireless sensor v.1.1; Champalimaud Scientific Hardware Platform) conveys motion information sampled at 200 hz (set on WEAR v.1.3.2 software; Champalimaud Scientific Hardware Platform) to a receiver base-station (Harp basestation v.1.1 or v.1.2, Assembly v.0, Harp v.1.4, firmware v.1.5; Champalimaud Scientific Hardware Platform), which conveys the information to the experimental computer running a Bonsai script (Bonsai^44^ editor v.2.3.1; RRID: SCR_017218) to capture and record motion data and video information. Video was captured using a camera (Flea3 FL3-U3-I3Y3M(17450451), Point Grey Research) coupled to a 1/2′′ format lens (NMV-6WA, Navitar). Open field with head-mount protocol deposited on protocols.io (10.17504/protocols.io.q26g7pnk9gwz/v1).

### Calibration

To ensure sensor stability within the sessions, several approaches were used. First, a coated mating sleeve was attached to the dual-optic fibre cannula that sits immediately posterior to the sensor. The sleeve was thickened with black tape to a desired outer diameter such that it stabilized the sensor in the anterior–posterior direction. Second, the metal pins in the four-position connector glued to the sensor were thickened with solder to stabilize their fit inside the receptacle connector in the skull cap. This protects against displacement in all directions. Third, stretchable black tape was wound around the base of the attached sensor and sleeve-covered cannula, further protecting against shifts in sensor positioning.

To control for possible variation in sensor positioning across sessions, a calibration approach was developed. A wireless inertial sensor was attached to individual isoflurane-anaesthetized mice and the sensor was secured using the above-described strategies. Next, individual mice were placed into a custom-made calibration rig. The essential element of the rig is a vertical stainless-steel pole suspended above a stably secured table. In the set-up used, the vertical pole was fixed to the horizontal edge of a vertically reversed L-shaped, stainless-steel post assembly mounted onto a breadboard (Thorlabs). The space between the lower end of the vertical pole and the table is enough for an individual mouse to slide underneath. The lower end of the vertical pole is fixed to a custom-made connector that resembles the connecting end of the fibreoptic patchcord. To perform calibration, individual isoflurane-anaesthetized mice was securely attached to the vertical pole through a mating sleeve bridging the connection to the mouse’s cannula implant. Next, replicate readings of the immobilized inertial sensor were made on Bonsai. Next, mice were attached to the experimental patchcord and allowed to recover in home cage for 20 min or until individual mice were clearly recovered and behaviourally active. Individual mice were then placed in open-field box for experimentation.

Calibration involves rotating all accelerometer and gyroscope readings from the inertial sensor by a rotation matrix such that the final gravitational field vector of the stationary sensor, when mounted onto the mouse and fixed to the calibration rig, is in a universal frame of reference whereby there is zero vertical tilt. In other words, the only non-zero acceleration is on the universal *z*-axis (pointing down). To accomplish this, the accelerometer pitch and roll orientation angles of the fixed stationary accelerometer were determined and then applied to calculate the rotation matrix. The rotation matrix is multiplied by the sensor accelerometer and gyroscope readings to remove the stationary vertical tilt from the sensor. To account for possible drift in gyroscope baseline over time, a daily reading of stationary gyroscope baseline was made with a mock cement skull cap attached to the sensor just before the start of each experimental day. The baseline gyroscope readings were subtracted from all gyroscope values before the rotation matrix is applied to sensor data.

### Action selection

After the open-field run in the grey-walled box, off-line behavioural clustering was performed on calibrated sensor data. To identify the natural action repertoire of individual mice, we quantified behaviour using acceleration and gyroscope time-series features in a similar manner to that described previously^21^. For the ground-truth analysis, we used: (1) gravitational acceleration along the anterior–posterior axis for the discrimination of postural changes, GAap. (2) Raw sensor acceleration along the dorsal–ventral axis to quantify movement momentum, ACCdv. (3) Dorsal–ventral axis of the gyroscope to extract head head–body rotational information, GYRdv. (4) Total body acceleration to differentiate the resting state from movement.

Total body acceleration (TotBA) was defined as:

$${\rm{TotBA}}={\rm{sqrt}}({{\rm{BAap}}}^{2}+{{\rm{BAml}}}^{2}+{{\rm{BAdv}}}^{2}),$$

where BAap, BAml and BAdv represent the body acceleration of the anterior–posterior, medial–lateral and dorsal–ventral axis, respectively. We calculated each individual BA component by median-filtering the raw acceleration signals followed by a fourth-order Butterworth high-pass (0.5 Hz) filter. For the gravitational acceleration axis, the BA components were subtracted from the median filtered raw signal axis.

All four time-series features were binned into non-overlapping 300 ms long window segments^45^. The values of each bin and per feature were then discretized, using fixed thresholds, producing a summary distribution of each segment. For GAap and ACCdv, we used ten equal size threshold values, plus two added bins between the limits and infinity to capture an approximated distribution of values within each window bin. For GYRdv, we used five thresholds (0, ±50, ±100) to discriminate left and right turns. For TotBA, a single threshold was used to separate moving from resting. The threshold was kept constant for all of the experiments and was set to the average value separating the bimodal distribution of log[TotBA] (natural logarithm of the TotBA feature). For each 300 ms window segment, we get four resulting histograms, one for each feature. The feature histograms were individually normalized to obtain probability distributions and were used to calculate the pairwise similarities between segments.

We used earth mover’s (EM) as a measure of similarity^27^:

$$S=-{({\rm{dEM}}/4)}^{2}$$

where dEM is the sum of the normalized EM distances for the four features (GAap, ACCdv, GYRdv and TotBA) defined above. The bin normalizations constrain *S* values within the range [−1,0], specifically, −1 and 0 define the maximum dissimilarity and identity between the two probability distributions, respectively. Finally, to produce a continuous unbiased classification of behavioural states, the similarity measures were clustered using affinity propagation^20^, with the preference parameter set to the minimal value of the similarity matrix; this particular value was used for its stable number of behavioural clusters within its range.

Using the behavioural clusters identified by affinity propagation clustering of the grey open-field behaviour as a ground truth for the true identity of each 300 ms histogram, we were able to simulate and evaluate the precision with which the EMD metric^21,27^ could be applied for cluster-matching online. A notable difference for the EMD metric used here is the use of the four features mentioned above rather than the three features used previously^21^, as well as the multiplication of the similarity score by −1 such that the range of possible scores from maximal identity to dissimilarity is 0 to 1, respectively. Although the EMD cluster-matching outcome correlates strongly with affinity propagation clustering, some false positive and false negatives may occur. Several filters were set to optimize cluster selection for reinforcement: (1) we selected for clusters that show low false-positive rate (<5.5%) and below the 60th percentile false-positive rate among all clusters per animal. (2) We selected against clusters with high false-negative rates (>90th percentile of clusters per animal). (3) We selected against clusters that tend to be performed serially within a short time interval. We calculated the probability that a target cluster or its top 5 most similar clusters (determined by EMD score) would reappear 3–18 s after the first occurrence of the target cluster. Clusters that tend to be repeated either by itself or have a high probability of having similar clusters appear within this 15 s window (>90th percentile for median and range of probabilities of cluster appearing in window) were removed from the selection pool. (4) We filtered against clusters of which matching by EMD would be more sensitive to anterior–posterior shifts of the inertial sensor (although we already protected against this possibility with the safeguards described above) (>90th percentile for percentage deviation from original cluster matching after shifts of accelerometer reading in the anterior or posterior direction). For each cluster, the percentage deviation is calculated first by summing the total absolute cluster-matching changes from the original cluster-matching data in the anterior and posterior shifted datasets. Next, the sum of deviation in the two altered datasets is divided by two and then divided by the total of cluster calls from the original dataset, and multiplied by 100 to get the percentage deviation from the original cluster-matching result. (5) We selected for clusters that show fully accelerating movement (cluster exemplar value of 1 (maximum value) in the body acceleration feature bin of histogram). We did not choose the exact same type of actions across animals to improve generalizability of discovered learning principles. However, action types comprise a mix of complex behaviour visually similar to those described previously^21^. To choose dissimilar clusters per animal, an algorithm was written filtering clusters of each animal’s repertoire based on the feature histogram values of each cluster’s representative, or exemplar. Thresholds were set along the GAap and GYRdv features to divide cluster exemplars based on the distribution of values within these feature histograms. For each repertoire, all histogram values from all cluster exemplars are pooled to create a pooled histogram. The range of bins with non-zero values for each feature are identified. The algorithm then filters cluster exemplars in the repertoire for non-zero values in the high, medium, low or high + low value bins. For example, action A identification occurs by selecting for a cluster exemplar with median counts falling in the high GAap and GYRdy value bins. Action B would then be selected by filtering for an exemplar with median counts falling in the low GAap and GYRdy value bins. This process was alternated from animal to animal, such that the selection criterion for action A and B would continually be reversed (for example, animal 1’s action A (high GAap/GYRdy values) and action B (low GAap/GYRdy values)→animal 2’s action A (low GAap/GYRdy values) and action B (high GAap/GYRdy values)→animal 3’s action A (low GAap/GYRdy values) and action B (high GAap/GYRdy values)→…). This results in action pairs that are highly dissimilar within animals and actions A and B that are broadly distributed across the action space. Dissimilar behaviours differ in complex ways, but visually can be roughly described as varying in some example dimensions such as vertical versus horizontal posture, heads down versus heads up and left versus right turns. EMD similarity scores comparing action A to action B almost always, except for 1 ChR2–YFP animal, fall at the more dissimilar end of a distribution of scores created by comparing action A to all actions in each animal. Hereafter, clusters are referred to as actions.

### Closed-loop optogenetics

For closed-loop optogenetics, a computer running a Bonsai script captured and recorded wireless sensor motion data and video information as described above in grey-walled open-field experiment. Here, data are also streamed to a custom MATLAB code that analyses action composition changes over the course of action reinforcement; we used the EMD metric^21^ to label individual 300 ms motion histograms with an action ID. For each arriving 300 ms segment, we calculate the EMD distance between each cluster exemplar (or representative) of the ground-truth cluster library from the grey open-field behaviour recording. The motion features histogram is assigned to the action for which comparison with the exemplar gave the lowest EMD score (most similar to the target action) among all comparisons. Decision making for stimulation has a time gap with a range of 35 to 55 ms between action performance and sent decision for stimulation. To trigger optogenetics, a multi-pulse width modulation (PWM) generator (Harp Multi-PWM Generator hardware v.1.1, Assembly v.1, Harp v.1.4, Firmware v.1.1; Harp Multi-PWM Generator software v.2.1.0; Champalimaud Scientific Platform) converts each decision to trigger laser into electrical signals for 15 light pulses of 10 ms pulse duration at 25 Hz, with each train of pulses occurring over 600 ms and at 25% duty cycle. The multi-PWM signal is passed through a 12 V, 7.2 W amplifier (Champalimaud Scientific Platform) and a fixed-frequency driver (Opto-electronic, MODA110-D4-30, 2001.320220) to control the activities of a 473 nm, blue low-noise laser (Shanghai Dream Lasers Technology, SDL-473-200T), which was sent through an acousto-optic modulator (Opto-electronic, MTS110-A3-V1S (1001/330433)). The laser component that is modulated is then reflected by a mirror and funnelled to a mono fibreoptic patchcord, which is then coupled to a commutator. The output laser is then passed through a dual-optic fibre patchcord and connected to the implant cannula. Power adjustment out of the tip of patchcord was made so that ~5 mW was emitted from each end of the dual-optic fibre cannula. To ensure common time stamps from different channels, a clock synchronization device (Harp Clock Sync v.1.0; Champalimaud Scientific Platform) was performed between the base station and multi-PWM device.

### Single-action sequence reinforcement

Mice were placed into a white open-field box for closed-loop reinforcement protocol. A white open-field was used instead of the earlier grey open field to minimize habituation effects, which would lead to reduced initial spontaneous behaviour during closed-loop protocol. Individual mice were subjected to a single session of the protocol each day, with sessions following each other on consecutive days. The white open-field box is made of 410 × 400 mm white matte acrylic walls and a 410 × 400 mm white matte acrylic base. To acquire the baseline behaviour, individual mice were allowed to behave freely inside the box for 30 min on the first action A reinforcement session. Closed-loop reinforcement by blue laser stimulation of VTA DA neurons was made available for 60 min. A total of 60 min of closed-loop reinforcement was made available for individual mice during sessions 2 and 3. For session 4, an extinction protocol was performed comprising 20 min maintenance of reinforced behaviour with laser availability, followed by 60 min of extinction of reinforced behaviour without laser availability, followed by 20 min reacquisition of reinforced behaviour with laser availability. To select for action B, a repeat of the protocol described above for action A was performed starting on the day after the extinction protocol for action A. After completing the reinforcement and extinction protocols for action B, a contingency degradation protocol was performed comprising 20 min maintenance of action B with laser availability, followed by 60 min of contingency degradation of reinforced behaviour by triggering the laser randomly, followed by 40 min reacquisition of reinforced behaviour with laser availability for action B performance. Protocol deposited on protocols.io (10.17504/protocols.io.4r3l221k4l1y/v1).

### Photometry experiment

One month after surgery, the mice were habituated to head-mounted equipment for 2 days. On day 1, habituation was made to wireless inertial sensor as described above. On day 2, a multifibre bundled patch cord (3 fibre bundle, 400/440 μm diameter for a maximum of inner diameter at 900 μm, 0.37 NA, 3.5 m long, 1.25 mm fibre tip diameter, low-autofluorescence; Doric, BBP(3)_400/440/900-0.37_3.5m_FCM-3xMF1.25_LAF) was attached to individual mice in addition to the wireless sensor and optogenetic patchcord. Individual mice were allowed to habituate to the equipment for 1 h in their home cage. On day of photometry recording, mice were subjected to 30 frames per second photometry recording (FP3002, Neurophotometrics), with a 75–150 μW 560 nm LED illuminating rDA1m, and equivalent closed-loop optogenetic parameters described above were used. To test for DA release in the context of the closed-loop optogenetic set-up, an average of 30 hits of blue light was delivered randomly within the span of 30 min. To evaluate DA release in the context of food reward, mice were placed on the food-deprivation protocol and kept within 85% of their original weight. Mice were placed into an operant chamber with a nosepoke linked to a lick detector (PyControl). Each lick detection triggers dispensing 2 μl 10% sucrose. As the animals tend to accidentally trigger the lick detector at the beginning of sessions, between 40 and 50 sucrose dispensing events were gathered per animal and rDA1m activities associated with the last 35 rewards of the session were used for analysis. Acquisition deposited on protocols.io (10.17504/protocols.io.bp2l6xz5dlqe/v1).

### Two-action sequence reinforcement

Two action sequence reinforcement occurs as follows: after sensor/patchcord habituation and grey open-field behaviour recording, offline behavioural clustering and action filtering were performed as described for single action reinforcement. For each animal, the median time intervals between all possible pairs of actions during open-field sessions were calculated as described above. Across animals, T1/T2 pairs with median T1→T2 interval values varying between 2 and 10 s were selected. To control for variation due to order of movement orientations, various combinations of target action T1 with low GAap/directional GYRdy values to target T2 having high GAap value/opposite directional GYRdy values were chosen for reinforcement.

On the first reinforcement session, a 30 min baseline was taken when laser stimulation was not available for reinforcement. The laser became available for reinforcement in all of the subsequent sessions until the extinction experiment. During the reinforcement periods, when the closed-loop system detects performance of the proximal action (T1) of interest, the algorithm enters a state in which the laser is triggered on performance of the distal action (T2), regardless of the amount of time that has elapsed between the latest T1 and T2. On session 1, 60 min of laser availability was given, while, in all of the subsequent reinforcement sessions, 90 min of laser availability was given.

### Histology and immunohistochemistry

After behavioural sessions were completed, mice were deeply anaesthetized with isoflurane and perfused transcardially in PBS and then 4% PFA/PBS. Dissected brains with skulls attached were perfused in 4% PFA in PBS at 4 °C overnight. The next day, the brains were rinsed three times in PBS. Next, brain regions including VTA and implants were sectioned by a vibratome into 50 or 100 μm slices. The slices were then analysed using immunohistochemistry (10.17504/protocols.io.eq2lyjbdelx9/v1) using the reagents listed below. Standard immunohistochemistry protocols were applied to stain for the following reagent: rabbit anti-GFP 488 conjugate (1:1,000; Molecular Probes, A21311); mouse anti-TH (1:5,000; Immunostar, Th 22941) with goat anti-mouse IgG (H + L); highly cross-adsorbed secondary antibodies Alexa Fluor647 (1:1,000; Thermo Fisher Scientific, A-21236), DAPI (1:1,000 of 20 mg ml^−1^ stock; Sigma-Aldrich, D9542).

### Imaging

The Zeiss Axio Imager M2 microscope was used to acquire brain section pictures. ×10 tiled images were taken through the relevant fluorescence channels. The M2 is equipped with a fast Colibri.7 LED illumination for excitation of fluorophores. Images were captured using a high-sensitivity monochromatic sCMOS camera (Hamamatsu Orca Flash 4.0 v2). The objective used for the images is a ZEISS Plan-ApoChromat ×10/0.45, which allows us to resolve up to 577 nm when using a wavelength of observation of 520 nm and it is fully corrected for chromatic and spherical aberrations. Implant locations were determined using standard mouse atlas^46^.

### Single-action reinforcement analyses

For target-action frequency analysis, we analysed frequencies within 25 min windows at four timepoints: baseline (before first reinforcement trigger), early (after first reinforcement trigger in session 1 (action A) or 5 (action B)), mid (after 2 min mark in session 2 (action A) or 6 (action B)), late (after 2 min mark in session 3 (action A) or 7 (action B)). For 3D action repertoire plots, baseline-normalized frequencies were plotted and actions of which the time series include NaN or infinity values were discarded from the plot (plotted actions: 509 out of 514 actions, 15 ChR2YFP animals (action A); 427 out of 443 actions, 13 ChR2YFP animals (action B); 355 out of 356 actions, 10 YFP animals (action A); 341 out of 356 actions, 10 YFP animals (action B)).

Three parameters were assessed for rapid behavioural adaptation after cumulative closed-loop reinforcements: latency between target A triggered reinforcements, target A frequency and average behavioural similarity to target A. Latency refers to the time interval between consecutive triggers. To calculate the latency parameter, the average latency between ten consecutive target A triggered reinforcements after a specified number of cumulative reinforcements was taken and then normalized to the average latency taken over the final ten baseline target A instances that in simulations would have triggered reinforcement. To calculate the frequency parameter, the frequency of target A triggered reinforcements over the course of 1 min after a specified number of cumulated reinforcements was taken and then normalized to frequency of the final ten baseline target A instances that in simulations would have triggered reinforcement. To calculate the behavioural similarity parameter, the average behavioural similarity (EMD score) to target A between ten consecutive target A triggered reinforcement events after a specified number of cumulated reinforcements was taken and then normalized to the corresponding value taken over the final ten baseline target A instances that in simulations would have triggered reinforcement.

### rDA1m fibre photometry analyses

To evaluate DA release in the context of food reward, the delta *F*/*F*_0_ signal was plotted for the rDA1m signal aligned to lick detection/reward trigger (Fig. 1f). The baseline *F*_0_ value was taken as the median rDA1m raw fluorescence signal of the ten timepoints (333.33 ms) preceding the trigger event. To test whether DA release is triggered in the context of the closed-loop system, the activity of the rDA1m sensor was quantified. Delta *F*/*F*_0_ was calculated by subtracting baseline value from each fluorescent rDA1m value of a smoothened time series (smooth function, default moving average filter, MATLAB), and then dividing the outcome by the baseline value. To account for control ChR2-independent effects, the average delta *F*/*F*_0_ trace of ChR2–YFP animals was subtracted from the corresponding average trace of YFP animals, giving the differential delta *F*/*F*_0_ used for the plots. The s.d. of ChR2–YFP minus YFP curves were obtained by taking the square root of the sum of squared variances of ChR2–YFP and YFP delta *F*/*F*_0_ curves.

### Categorizing behavioural actions by temporal dynamics

To categorize behavioural actions by temporal dynamics (Fig. 1m and Extended Data Fig. 6), the moving mean of action counts was used as an input. Various window sizes were examined; 2.5 min windows moving at 300 ms steps were found to be suitable for analyses. The baseline frequency (*f*_0_) was the average of 5 min of moving mean data preceding the first reinforcement event. The early frequency rate (*f*_1_) was the average of 30 min moving means immediately after the first reinforcement event. The mid- and late frequency rates were taken from day 2 (*f*_2_) and day 3 (*f*_3_), respectively. *f*_2_ and *f*_3_ rates were calculated from the beginning 30 min period after moving windows had accumulated enough bins (2.5 min) after the start of the session. Significant positive modulation above baseline was judged if, in 500 consecutive moving windows (2.5 min period) in the early/mid or late stages, the frequency rate of all bins was greater than the 99th percentile bin of the baseline frequency. Significant negative modulation below the baseline was judged if, in 500 consecutive moving windows (2.5 min period) in the early/mid or late stages, the frequency rate of all of the bins was less than or equal to the 5th percentile bin of the baseline frequency. Actions that showed both significantly positive and negative modulation at the early/mid or late stages when compared to the baseline were delegated to the positive modulation group. For figure plotting, time-course median frequencies of action dynamic types were downsampled tenfold (Extended Data Fig. 7a). To examine the distribution of action dynamic type frequencies in terms of target similarity, a binning by raw EMD score (0.5 score binwidth) was used because this allowed for clear visualization of the relationship between target similarity and frequency (Extended Data Fig. 7b). Alternatively, percentile binning of the EMD score was also used and gave similar trends (Extended Data Fig. 7c).

### Criterion for action dynamic types

Action dynamics were grouped as follows: (1) increasing actions showed a significant increase in *f*_0_ to *f*_1/2_ and *f*_1_ to *f*_2/3_ comparisons and showed either a significant increase or unchanged frequency in *f*_1/2_ to *f*_3_ comparisons. (2) Sustained actions showed a significant increase in *f*_0_ to *f*_1/2_ comparisons, and an unchanged frequency in *f*_1_ to *f*_2/3_ and *f*_1/2_ to *f*_3_ comparisons. (3) Transient actions showed a significant increase in *f*_0_ to *f*_1/2_ comparisons, and a significant decrease in *f*_1/2_ to *f*_3_ comparisons. (4) Decreasing actions showed a significant decrease in *f*_0_ to *f*_1/2_ and *f*_0_ to *f*_3_ comparisons. (5) Other actions were all of the remaining actions that did not fall into the above groups. In Extended Data Fig. 7a, only dynamic subtypes with more than ten members are shown.

### *t*-SNE and hypervolume analyses

Data from 5 min portions of baseline (session 1 pre-reinforcement portion), early (session 1 reinforcement portion), mid (session 2) and late (session 3) were pooled to calculate the pairwise EMD similarities between all individual action instances, creating a similarity matrix. An individual action instance’s similarity distance against all other actions in the dataset specify the action’s position in behavioural space. The first 50 principal components of this dataset were embedded into a 2D behavioural space with *t*-SNE. Hypervolume analysis was performed using the dynamic range boxes method^36^, and implemented using the dynRB package in R (RRID: SCR_001905). To account for more information in *n*-dimensional space, the first 250 principal components of the similarity matrix (4,000 total action instances randomly subsampled in equal numbers from each baseline/reinforcement portion) were used to calculate hypervolume overlaps. The port parameter (the proportion of a particular action cluster’s hypervolume overlapping with that of the target cluster hypervolume) was evaluated. To evaluate changes to transiently increased dynamic type percentage per animal after accounting for overlapping hypervolumes, all action clusters of which the hypervolumes had non-zero overlap with the target hypervolume were removed to recalculate the percentages of each dynamics type per animal. Data are presented in Extended Data Fig. 8.

### Extinction analyses

**Extended Data Fig. 4:**
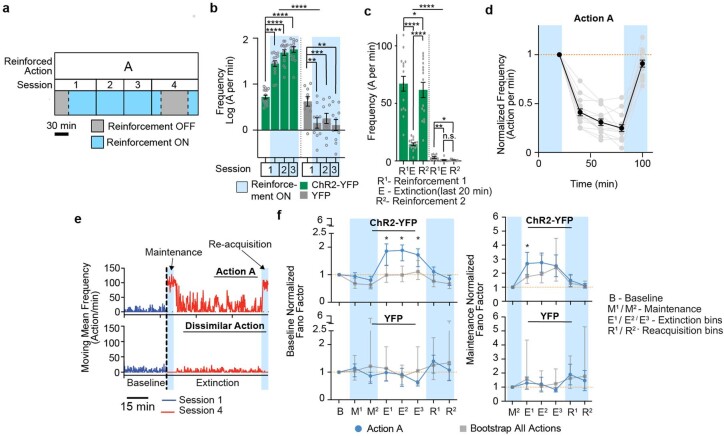
Reinforcement of action A in ChR2-YFP and YFP animals. **a-d**, *n* = 15 ChR2-YFP, 10 YFP animals (biologically independent). Plots in (**b-d**) show mean/S.E.M. **a**, Protocol for Action A reinforcement. **b-c**. Repeated measures two-way ANOVA reveal significant difference across time and ChR2-YFP/YFP groups in reinforcement sessions (F(3,69) = 82.61, p < 0.0001) and extinction session (F_2,46_ = 18.66, *P* < 0.0001). Tukey’s post hoc comparisons. **d**, Detailed extinction of action A reinforcement showing mean (black) and individual curves (light grey lines). Plot shows means/S.E.M. Asterisks: **** p < 0.0001. *** p < 0.001. ** p < 0.01. * p < 0.05. n.s. – not significant. **e**, Bursty target action behaviour upon extinction. Plots show moving mean frequencies of action A (target action) and a dissimilar action of an ChR2-YFP animal over baseline and extinction conditions. The dissimilar action is later reinforced for and is referred to as action B in subsequent protocols. **f**, Normalized Fano factor over extinction conditions (action A). Plots were mean/95% confidence intervals. *n* = 15 ChR2-YFP, 9 YFP animals. * - Mean of ChR2-YFP animals outside bootstrapped 95% confidence intervals; 2-tailed comparison. See Supplementary Information for statistical/sample details. Source Data

10 min portions from different time windows along the extinction protocols (session 4 for action A and session 8 for action B) were chosen (Extended Data Figs. 4c (action A) and 9g (action B)). Early maintenance (M^1^) starts from the first instance of target action performance in the session. Late maintenance (M^2^) is the portion preceding the first performance of the target action after extinction. Early extinction (E^1^) begins at the first instance of target action performance after extinction. Late extinction (E^3^) is the portion preceding the first performance of the target action after reacquisition. Mid extinction (E^2^) begins at the midpoint between the starts of E^1^ and E^3^. Early reacquisition (R^1^) starts at the first performance of the target action after the reacquisition condition. Late reacquisition (R^2^) is the final portion of the extinction protocol.

### Action burstiness analysis

To evaluate action burstiness, or dispersion, we used Fano factor (variance/mean) as a measure (Extended Data Fig. 4f). A survey of moving-mean frequencies of reinforced actions across animals suggest that actions are more dispersed during the extinction phase, but the timescale with which this may occur is variable. To identify a suitable timescale to detect dispersion across reinforced actions, we screened a range of window sizes (600 ms to 5 min windows in 600 ms steps) with which to calculate moving-window frequencies, and then calculate the Fano factor in varying time segments. We chose a moving window of 15 s (50 × 300 ms action units) to construct moving-mean frequencies. This window size consistently gave a decreased Fano factor in baseline versus maintenance session across animals, a result that would be expected as reinforcement led to stable target action performance.

### Single-action reinforcement, inter-target and inter-action interval analyses

To quantity inter-target action intervals (Fig. 3a,b), the median amount of time that transpired between the start of successive target actions over the course of a time window was calculated. The time periods analysed were: (1) baseline from the start of day 1 (sessions 1 and 5 for action A and B, respectively) until the first reinforcement event. (2–4) Days 1 to 3 reinforcement. For reinforcement periods, behaviour from the start of the first reinforcement event of that session until the end of session was analysed. We considered the possibility that including the time interval between consecutive repeating of target actions (resulting in an inter-target action interval of 300 ms) would greatly affect the result. To test this, we removed values collected from consecutively repeating target actions. However, this did not affect result interpretations. Thus, we included intervals from consecutively repeating target actions in the presented analyses. For single-action reinforcement, the median amount of time between the closest occurring action of interest and target action was calculated for both pre-target and post-target intervals.

### Multinomial logistic regression predicting action dynamic types

To test whether intrinsic and baseline action properties are predictive of classifiable action dynamics during single-action reinforcement from a naive state, two factors were considered. The factors are EMD similarity of action to target and median time interval of closest action of interest preceding target appearance at the baseline condition (Fig. 3c–g).

To perform multinomial logistic regression, data from both dependent variables were normalized to *z* scores. Transformed data were tested for collinearity by examining scatter plots, Pearson’s correlation coefficients, variance inflation factors (VIFs) and condition indices. The two variables showed some correlation, but the coefficient value was not above typical thresholds^47,48^ and direct collinearity diagnostics did not show significant collinearity (Pearson’s correlation: 0.61 < 0.8 (ref. ^47^), VIFs: 1.6 < 5–10 (ref. ^49^), condition indices: 2.0 < 10–30 (ref. ^50^). Multinomial logistic regression was performed using the MATLAB functions mnrfit and mnrval. The mnrfit function uses the iteratively weighted least-squares algorithm to find the maximum likelihood estimate of the coefficients in a multinomial logit model. In such a model, the relative risk of being in one action dynamic type category versus the reference group (decreased dynamics type) is expressed as a linear combination of predictor variables, each with its own *β*-coefficient. The mnrval function predicts the category probabilities. Non-target A actions from all animals from reinforcement of action A were included except those for which reinforcement dynamics were previously classified as ‘other’ types (*n* = 30 actions from a total of 514 actions, 15 ChR2–YFP animals). Model accuracies were assessed using a 20-repeat, 10-fold cross-validation approach for a total of 200 unique models for real data, and 10,000 unique models from 50 shuffled datasets.

To evaluate multinomial logistic regression, the deviance measure was used to judge model fitting (Fig. 3f). The deviance of the fit is a goodness-of-fit statistic that is calculated as twice the difference between the maximum achievable log likelihood (in a saturated model in which data fit perfectly) and the corresponding likelihood in the fitted model (the actual model of interest).

Model performances were judged by area under precision-recall curve as this criterion is suitable for imbalanced categories in the data^51^ (Fig. 3g). A model containing both dependent variables was found to outperform that of any single variable in data fitting, even after consideration for penalties for an extra factor (Akaike information criterion; 2 multiplied by 2 independent variables (2k = 4) as penalty added to deviance of two-factor model^52,53^. Furthermore, the two-factor model outperformed both one-factor models in predicting true positives of decreased type, while performing just as well as the similarity factor model in predicting true positives of sustain increased and transient types (Supplementary Table 1). The two-factor model outperformed the time preceding target factor model in predicting all types (see the ‘Fig. 3f(III)’ tab in Supplementary Table 1). The lack of significant collinearity between dependent variables was supported by the stability of two relevant parameters, *β*-coefficient directions and significant *P* values, across 200 cross-validation models and single- and double-factor regression conditions (tables are provided in the Supplementary Information).

### DA retrospective window analysis

To test whether reinforcement was selecting for behaviour before, during or after stimulation, we originally tracked how initial baseline distributions of single actions surrounding stimulation relate to single-action frequencies after reinforcement. However, we were not able to tell whether single-action frequency changes after reinforcement were associated with each action’s baseline distribution before or after stimulation (the distribution was highly symmetric due to lack of contextualization).

To improve contextualization, we analysed first-order transitions. This provided enough context for us to distinguish between behaviour occurring before, during or after stimulation. Thus, we were able to identify action transitions specifically associated with specific temporal positions relative to stimulation, track these action transitions as reinforcement commences, and attribute their frequency changes to their temporal positioning surrounding stimulation.

To analyse whether DA reinforces actions proximal to the target action, rates of action transitions occurring close to the reinforced action were examined during the baseline and over the course of closed-loop reinforcement (Fig. 3h–j). First, 600 ms action transition events (for example, X→Y) occurring from 2.4 s before to 2.4 s after each theoretical target-triggered laser stimulation (600 ms in length) during the baseline condition were examined. Next, all of the possible transitions occurring within specific 600 ms sliding windows within the defined time range were counted for each animal. Next, the relative performance of each action transition type in a specific sliding window against all of the sliding windows was calculated by dividing a particular action transition type’s count within a specific sliding window by the total number of the same action transition type across sliding windows. This gives probabilities across sliding windows. Next, action transition probability within a sliding 1.2 s window (Fig. 3h (within); containing a total of three action transitions) relative to surrounding temporal environment (Fig. 3h (outside); 3.6 s) was derived by subtracting the probability of a particular action transitions type occurring inside an outside region from the probability of the same type occurring in the within region of interest. This will be called the differential probability. Next, action transition types that showed greater or equal to a threshold of 0.001 differential probability within sliding 1.2 s windows of interest over the corresponding surrounding outside windows were filtered and kept for the next step. This marks the selection for enriched action transitions. Next, for each sliding 1.2 s window, transition count data from above were analysed to select for action transition types that occurred between 2 to 6 times during the 30 min baseline period (0.067 to 0.2 action transitions per minute). The count range was chosen to filter out single events while selecting for action transitions with low initial frequencies over the baseline period and analysis time range. As the range of probabilities of specific action transition types could vary greatly between different sliding 1.2 s windows, filtering as described above also balances the distribution of action transition probabilities among all action transition types analysed across sliding 1.2 s windows. The above process results in a list of action transition types enriched for each sliding 1.2 s window, and baseline-normalized frequencies of these action transition types after reinforcement in subsequent sessions were calculated. Note that baseline-normalized frequencies were calculated from all occurrences of specific action transition types, regardless of their time distance in relationship to target occurrence. Baseline-normalized frequencies of individual action transition types were averaged within animals and the means between animals were averaged to produce animal-balanced results (Fig. 3i,j). Identical data trends and conclusions could be reached even if baseline-normalized frequencies of all action transitions were used for analyses.

### Two-action sequence experiment analyses

Two-action sequence frequency was quantified in terms of laser triggers per minute (Fig. 4b,d–g). To assess learning across animals, the baseline frequency was subtracted from frequencies of all reinforcement sessions (Fig. 4b,d). A criterion baseline-subtracted frequency of 3.2 triggers per minute was set after considering the range of baseline-subtracted frequencies observed in the open-field and reinforcement sessions for all animals. The criterion is set such that it is >20% above the highest baseline-subtracted frequency value seen in the open-field condition. The criterion point consistently falls above the open-field frequencies of all animals and marks the rising phase of all reinforcement frequency curves.

T1→T2 intervals were quantified as the time distance between the end of the latest distal action (T1) and the end of the proximal action (T2) that triggers the laser. T2→T1 intervals were quantified as the time distance between the end of T2 that triggers laser and the end of the next closest T1. To produce equivalent measures in open-field and baseline conditions, laser trigger events were simulated by scanning across the data as if reinforcement was available.

Significance testing was performed on 14 out of 15 ChR2–YFP animals that reached the criterion frequency (ChR2–YFP criterion) (Fig. 4e). The lone animal that did not reach the criterion frequency was removed because the T1→T2 median interval was still very high after session 10. This animal was subsequently subjected to single-action reinforcement protocol to assess its ability to learn T1 and subsequently T2 (Extended Data Fig. 10a,b). Next, the animal was again subjected to the T1→T2 reinforcement protocol (Extended Data Fig. 10c). These results indicate that this animal was capable of action learning for both T1 and T2 separately, and for T1→T2 sequence after learning of each individual action.

Reinforcement sessions for the 14 ChR2–YFP animals that reached beyond the criterion frequency continued until the T1→T2 interval had been decreased to below at least a median of 2 s (Fig. 4h). As YFP animals do not decrease the T1→T2 median interval over sessions, we stopped reinforcement at session 20.

### Two-action sequence extinction

In total, 14 out of 15 ChR2–YFP mice that passed the criterion sequence frequency were subjected to the following extinction protocol. The remaining animal was subjected to an earlier extinction protocol spaced over two sessions and had a longer stretch of extinction time (Extended Data Fig. 10). This animal showed a loss of sequence performance over the extinction period but became largely inactive by the reacquisition period, slowing reacquisition within the allocated time window. This led to shortening of the extinction protocol to 40 min of extinction and having all conditions within a single session as described here. The extinction session begins with a 25 min maintenance period for two-action sequence reinforcement, followed by a 40 min extinction period during which the laser was inactive, followed by a 25 min reacquisition period whereby reinforcement was made available again (Fig. 4f,g). To quantify performance for plotting, the frequency was calculated over 5 min bins and then normalized to the last 5 min bin of the maintenance condition (Fig. 4f). For significant testing, raw frequencies were analysed at the last 5 min of maintenance, extinction and reacquisition conditions (Fig. 4g).

### Two-action sequence refinement

To measure refinement for T1 and T2 in the two-action sequence (Figs. 4k and 5), actions that were uniquely related to one but not the other were identified. Actions performed by each animal in their open-field repertoires were ranked by their EMD similarity scores to T1 or T2. The top-12 actions (within action repertoires ranging between 30 and 40 actions) most similar to either T1 or T2 were identified. Actions common to both T1 and T2 in these lists were removed, leaving actions uniquely similar to T1 or T2. We required at least three non-target actions to be uniquely related to each of T1 and T2. One of the animals did not meet this requirement, because less than three actions were uniquely similar to each of T1 and T2 when considering the top-twelve actions related to T1 or T2. For this animal, we relaxed the stringency by considering actions that uniquely belong as the top-nine actions most similar to either T1 or T2. We took the median target-normalized frequency of these uniquely similar actions to T1 or T2 as the refinement index. A refinement index of above or around 1 indicates little to no refinement of uniquely related actions to target. A refinement index of below 1 indicates refinement relative to target; the lower the score, the more refinement. Refinement curves were smoothened using the Savitzky–Golay filter to improve visualization of trends (Fig. 5a). To better compare the progress of refinement between T1- and T2-related actions, refinement indices were scaled such that the minimum value among all sessions for individual animals would be zero and the target-normalized median frequency of 1 would remain at a scaled value of 1 (Fig. 5a,f).

### The relationship between T1→T2 interval and sessions to criterion frequency

To describe the trend in a T1→T2 interval versus sessions to criterion frequency scatter plot, nonlinear sigmoidal fit was tested against a fourth-order polynomial fit (Fig. 4l). A linear fit was also tested. Sigmoidal fitting gave the best result. The same fitting was tested for T2 → T1 interval versus sessions to criterion frequency, but the fit was poor, and the midpoint was unstable. For the T1→T2 sigmoidal curve, the half-maximum was 2.59 sessions to criterion frequency and the midpoint was 4.69 s of open-field median interval. The half-maximum value was used to divide ChR2–YFP mice into slow (above half-maximum) and fast (below half-maximum) learners. Identical grouping of fast and slow learners could be obtained by taking the median value across animals as the separation point.

To test whether reinforced action pairs differ in initial inter-trigger intervals (Extended Data Fig. 11d), we simulated and calculated the natural median interval occurring between T1→T2 performances (called the median inter-trigger (simulated) interval) and examined whether variation in this parameter at the baseline predicts the learning outcome (sessions to criterion frequency). To test whether the initial T1→T2 median interval influences learning after matching for reinforcement numbers over learning (Extended Data Fig. 11e–g), learning was examined by calculating the average number of sequence performances per unit time, with the time range covering spans of 200 reinforcements. To further account for differing initial and eventual sequence frequency over time, we smoothed all individual learning curves with a Savitzky–Golay filter and then scaled the curves to the frequency in the initial 200 reinforcement bin (value = 0) and to the maximal frequency (value = 1). To ensure that a similar conclusion regarding the relationship between initial T1→T2 intervals and learning could be derived, we plotted initial T1→T2 median intervals against the number of reinforcements cumulated after reaching the criterion frequency (25% of maximal scaled frequency) and tested for the ability to fit a sigmoidal curve as in Fig. 4l.

### Differential refinement analyses

The difference in area between T1 and T2 scaled refinement curves over sessions was used to assess the relative refinement status between T1 and T2 over sequence learning (Fig. 5b). The difference in areas was summed up using the trapezoid method across sessions until the session when both T1 and T2 has or had reached minimal scaled refinement. Next, the relationship between the open-field median interval and average difference in area under T1–T2 refinement curves per session was tested. A per-session metric was used to control for variations in total sessions across animals. Linear regression proved to be most suitable for fitting (goodness of fit: *R*^2^ = 0.66). The fit for the T1→T2 linear line was *y* = 0.1893*x* − 0.7050. The slope was significantly non-zero (*P* = 0.0004). The same fitting was tested for T2→T1 interval versus difference in area under T1–T2 refinement curves per session (*y* = 0.00736*x* + 0.1356), but the fit was poor, and the goodness of fit was low (goodness of fit: *R*^2^ = 0.07). The slope was not significantly non-zero (*P* = 0.7063).

It is possible that, for fast learners, the differential refinement favouring proximal action (T2) is seen only briefly in very early parts of learning (for example, intrasession blocks within sessions 1 and 2). To account for this, we probed intrasession refinement dynamics for fast learners by dividing each session into three 30 min blocks and repeated the refinement analyses in Fig. 5b (Extended Data Fig. 13a). We performed the analysis only up to the session of criterion frequency (usually one of the early sessions for fast learners) to avoid diluting out the effect from later sessions when differential refinement tends to even out as animals learn the sequence. We further visualized differential refinement dynamics for fast learners similarly to Fig. 5c.

The intrasession analysis showed that fast learners underwent diverse differential refinement dynamics. Some fast learners show differential refinement within the session for the proximal action (T2), whereas others show the opposite (favouring T1 early on). Some did not show clear preference for either. Importantly, these higher-resolution results were consistent with the analyses done based on whole-session data (Fig. 5b). A similar linear relationship between open-field T1→T2 median interval and differential refinement (change in area under the T1–T2 refinement curves (per time block)) was observed (*R*^2^ is 0.65 and non-zero slope is significant at *P* < 0.001 for the within-session data plot).

### Starting-point identification for evaluating progression of differential T1/T2 refinement

To more precisely examine whether proximal action (T2) refinement precedes that of distal action (T1) in slow learners, it was important to consider refinement progression of T1 relative to T2. To rule out any bias towards proximal refinement because of initial bias towards proximal T2 refinement, a specific session was chosen as a starting point for the analysis for each animal (Fig. 5c). This starting point was defined by an early session in which T1 and T2 were relatively similar in refinement levels or when the distal action T1 was more refined than proximal T2. To identify these starting points, a scan was made retrospective from the session for which the T1→T2 time interval is close to final value (less than or equal to a median of 3 s). Using this approach, we identified earlier sessions in which distal T1 refinement was equal to or greater than proximal T2 (T2 − T1 refinement curve area less than or equal to 0). The latest such session was set as the starting point for analysis. If at no point early in learning did an animal have a session in which the proximal (T1) action was most refined relative to the distal (T2) action, an early session of closest T1 and T2 refinement was used as the starting point. The initial T2 − T1 refinement curve area difference calculated from the starting point to next session was subtracted from all T2 − T1 area differences calculated in subsequent sessions. This value is called the starting-point-subtracted refinement difference (Fig. 5c). This made it possible to clearly track the change in relative refinement of distal (T1) versus proximal (T2) actions over time (values above zero indicate T2 > T1 refinement, and values below zero indicate T1 > T2 refinement). To identify the turning points for each animal, sessions carrying the local maximum value of the starting-point-subtracted refinement difference were identified for each animal. To calculate the starting-point-subtracted refinement, scaled refinement values from sessions of interest were subtracted from that of the starting point session defined above (Fig. 5g).

### Odds-ratio analysis

For odds-ratio calculation (Fig. 5e), the total relative change in T1→T2 and T2→T1 median intervals values were analysed for two different phases of learning: (1) the sessions from open-field to turning point session (open-field→turning point), and (2) the sessions from turning point session to the session reaching criterion frequency (turning point→session reaching criterion frequency). The total change in median intervals from open-field to session of criterion frequency was summed up for each interval type, using the change in values from open-field→turning point and turning point→session reaching criterion frequency. Next, the proportion of the total interval change stemming from the open-field condition→turning point period, and from turning point→session reaching criterion frequency period, was calculated. Next, the proportion of open-field→turning point interval change was divided by the proportion of turning point→session reaching criterion frequency period interval change for T1→T2 and T2→T1 interval types, respectively. This gives the odds ratio.

### T1 probability rank changes across time bins from the T2 trigger

For every actual or simulated trigger for T1→T2 performance, the first occurrences of every action before or after T2 triggers were counted at specific 300 ms time bins for up to 6 s before and after the T2 trigger (Fig. 5h). This was done for the specific conditions of the baseline, starting point, turning point, session passing criterion frequency and last session. The probability of an action occurring in a specific 300 ms time bin was calculated for all actions in the repertoire, and the values were used to determine probability rank in terms of percentiles (100 percentile is most probable action relative to all actions at a specific 300 ms time bin). To assess the total T1 probability rank change within 0.3–1.8 s or 2.1–3.6 s time bins, the area under the curve was determined and values were normalized by subtraction from each animal’s corresponding baseline values.

### Statistical analysis

Standard statistical analyses were performed using Prism (v.7, v.9, v.10; GraphPad Software; RRID: SCR_002798) and permutation/bootstrap analyses were performed in MATLAB (MathWorks; RRID: SCR_001622). To determine appropriate tests for comparisons, datasets were assessed for normality using Anderson–Darling, D’Agostino–Pearson, Shapiro–Wilk and/or Kolmogorov–Smirnov tests whenever applicable. Datasets were also visualized for normality using *QQ* plots and assessed for equal variance by examining the residual plot (residuals versus predicted Y). Parametric or nonparametric tests were chosen on the basis of the combination of these analyses. Data were transformed logarithmically (with or without addition of a constant before transformation) whenever it was appropriate to promote normality and equal variance. Unless specified, sphericity was not assumed, and Geisser–Greenhouse correction was applied in all ANOVA tests. The appropriate post hoc multiple-comparison tests were applied to compare between the means of specific conditions wherever applicable. Significance was set at *α* = 0.05. For bootstrap analysis, significance was determined by asking whether the original target action mean Fano factor was greater or less than the 95% confidence interval of the bootstrap distribution. Permutation tests were applied in the comparisons between regression models owing to the large sample size discrepancy between groups. Bonferroni *P* adjustment was used to account for multiple comparisons in this case. When GraphPad Prism does not output exact *P* values, Excel (v.16.78.3; Microsoft) was used with the ANOVA-specific FDIST(F, DFN, DFD) where DFN is the numerator degrees of freedom and DFD is the denominator degrees of freedom. Detailed descriptions of the statistical procedures are provided in the Supplementary Information.

All materials, methods, code and software used are available at Zenodo (https://zenodo.org/records/13249956).

### Reporting summary

Further information on research design is available in the Nature Portfolio Reporting Summary linked to this article.

## Online content

Any methods, additional references, Nature Portfolio reporting summaries, source data, extended data, supplementary information, acknowledgements, peer review information; details of author contributions and competing interests; and statements of data and code availability are available at 10.1038/s41586-023-06941-5.

## Supplementary information

Supplementary Methods Methods pertaining to the Article.

Reporting Summary

Supplementary Notes Notes of relevance to the Article.

Supplementary Table 1 Statistical summary of results in the Article.

Supplementary Video 1 Example of an action cluster performed on 10 instances in a grey open field box, before closed-loop reinforcement. Slowed down 3×.

## Source data

Source Data Fig. 1

Source Data Fig. 2

Source Data Fig. 3

Source Data Fig. 4

Source Data Fig. 5

Source Data Extended Data Fig. 1

Source Data Extended Data Fig. 2

Source Data Extended Data Fig. 3

Source Data Extended Data Fig. 4

**Extended Data Fig. 5:**
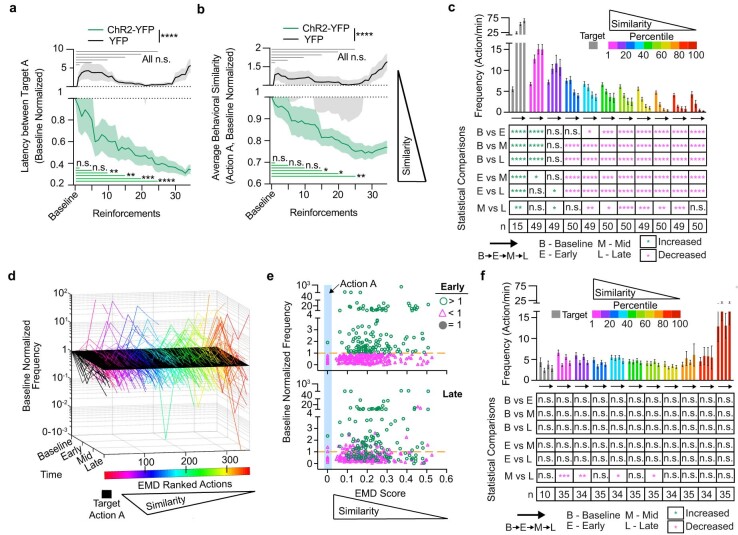
Reinforcement of Action A in ChR2-YFP and YFP animals. **a-f**, *n* = 15 ChR2-YFP, 10 YFP animals (biological replicates). Plots in (**a-c, f**) show mean/S.E.M. **a-b**, Rapid behavioural adjustments to close-loop reinforcements. Repeated-Measures 2way ANOVA, posthoc 2-sided Sidak’s multiple comparisons test: Significant Time x Group Interactions (F(35, 805) = 3.019, p < 0.0001 (**a**); F(35, 805) = 3.086, p < 0.0001 (**b**)). **c**, Expanded graph from Fig. 1 showing quantification of raw frequency changes across learning stage and target similarity percentile groups. Additional table below graph indicate 2-tailed, Tukey multiple comparisons results. *n* – sample size (actions). **d**, Evolution of pooled action repertoire (*n* = 355 actions from 10 YFP animals) across action learning from a naïve state. A black parallelogram was set at baseline normalized frequency = 1 to help visualization. **e**, Cross-sectional view of action repertoire frequencies at Early and Late stages. **f**, Same as (**c**) but for YFP animals. Asterisks: **** p < 0.0001. *** p < 0.001. ** p < 0.01. * p < 0.05. n.s. – not significant. See Supplementary Information for statistical/sample details. Source Data

Source Data Extended Data Fig. 5

**Extended Data Fig. 6:**
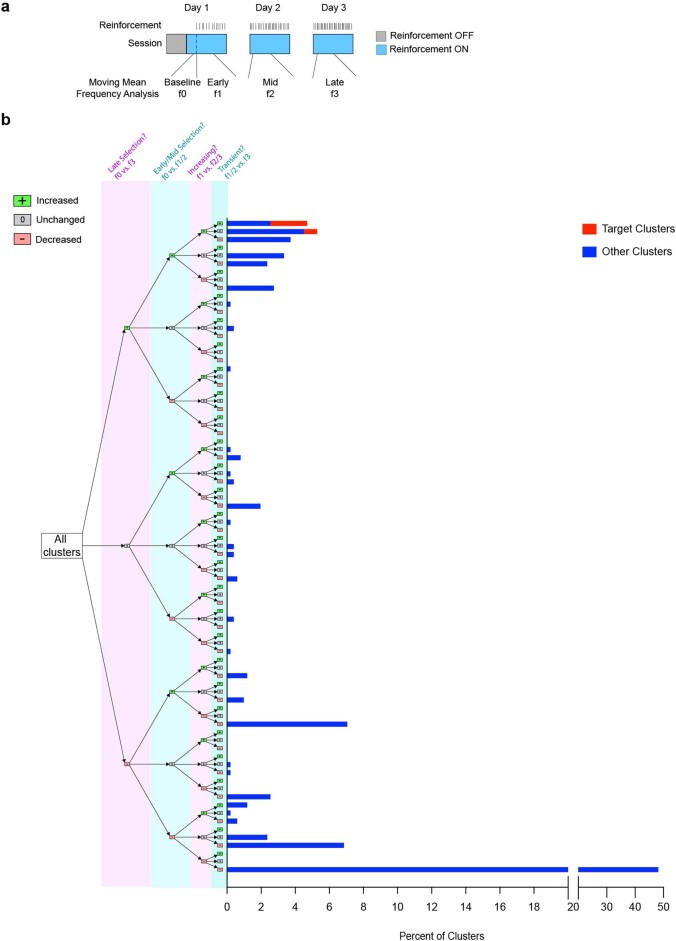
Classification of action dynamic types upon Action A reinforcement. **a**, Schematic of reinforcement protocol and definition of learning stages. f0, f1, f2 and f3 represent the moving mean frequencies from Baseline, Early, Mid and Late stages, respectively. **b**, Distribution of all actions according to criterion. 4 criteria were used. First, actions were divided by their final modulation status (Late Selection? f0 vs. f3). The next division is based on whether they were significantly modulated in early stages (Early/Mid Selection? f0 vs. f1/2). The third division is based on comparing initial modulation status in Early stage with later stages (Increasing? f1 vs. f2/3). The final division is based on whether actions in early stages decrease significantly by the Late stage (Transient? f1/2 vs. f3). Distribution across all possible combination of these comparison outcomes is plotted in terms of Percent of All Actions on the x-axis. *n* = 511 actions (496 other actions (blue bars), 15 target Actions A (red bars)), 15 ChR2-YFP animals (biological replicates). Source Data

Source Data Extended Data Fig. 6

**Extended Data Fig. 7:**
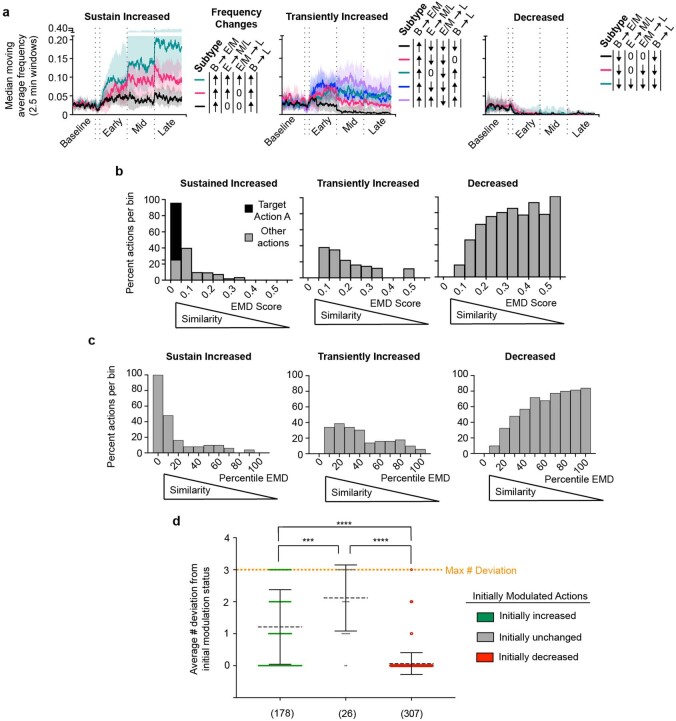
Classified Action Dynamic types and analyses. **a-d**, *n* = 15 ChR2-YFP, 10 YFP animals (biological replicates). Plots in (**d**) show mean/S.D. **a**, Three defined types of action dynamics. Plots were median moving average frequency of subtypes (lines) and 25th-75th percentile ranges (colour filled). Significant modulation: +, increased. −, decreased. 0, unchanged. **b**, Distribution of action dynamic types shift according to their percentile similarity to target. Histograms show percent clusters per bin vs. target action similarity. **c**, Distribution of action dynamics types shift according to action similarity to target. **d**, Initially decreased actions tend to retain its decreasing or decreased status throughout learning. Plot shows mean/s.t.d. (Kruskal-Wallis test, medians vary significantly, p < 0.0001. Kruskal-Wallis Statistic 237.8. 2-tailed Dunn’s post hoc tests: Initially increased (n = 178 actions) vs. decreased (*n* = 307 actions) – p < 0.0001. Initially increased vs. unchanged (n = 26 actions) – p = 0.0004. Initially decreased vs. unchanged – p < 0.0001). Asterisks: **** p < 0.0001. *** p < 0.001. ** p < 0.01. * p < 0.05. n.s. not significant. See Supplementary Information for details. Source Data

Source Data Extended Data Fig. 7

**Extended Data Fig. 8:**
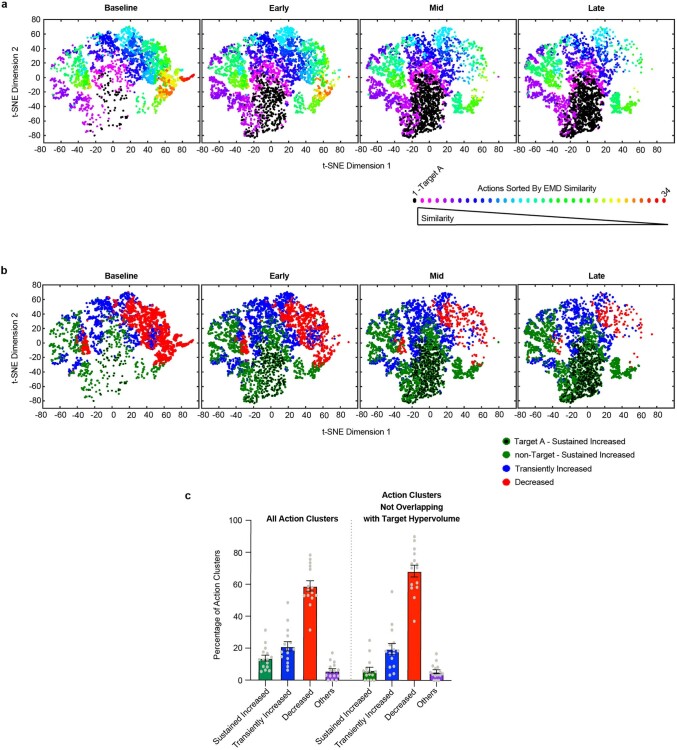
t-SNE and hypervolume analysis supplementing Fig. 1. **a-b**, t-SNE representing the evolution of actions over learning to perform Target A. Shown is the behavioural evolution of a representative ChR2-YFP animal. Actions clusters are colour labelled according to Earth Mover’s Distance (EMD) similarity to target action in (**a**) and according to action dynamics type in (**b**) (34 action clusters in total). Dots represent single action instances. Black dots (**a**) and black dots with green outlines (**b**) represent single instances of Target A. **c**, Percentage of classified action dynamic types based on all action clusters (left set of bars) or action clusters whose hypervolume did not overlap with target hypervolume (right set of bars). Bar graphs indicate mean +/− S.E.M. *n* = 15 ChR2-YFP animals (biological replicates). Source Data

Source Data Extended Data Fig. 8

**Extended Data Fig. 9:**
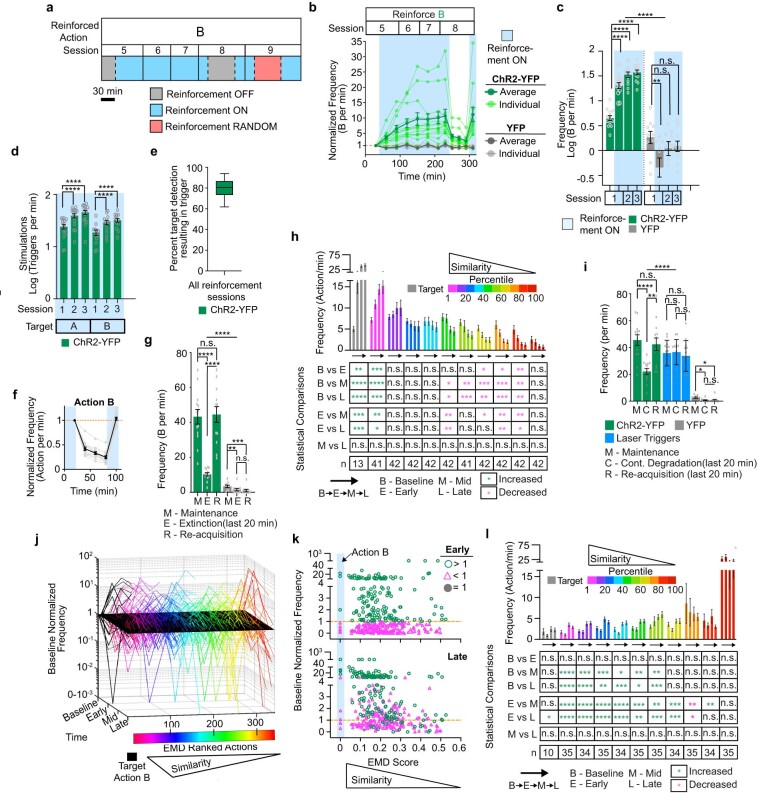
Closed-loop reinforcement of Action B in ChR2-YFP and YFP animals. **a-l**, *n* = 13 ChR2-YFP (Action B), 15 ChR2-YFP (Action A), 10 YFP (Action B) animals (biological replicates). All bar and line plots (**b-d, f-i, l**) show mean/S.E.M. **a**, Protocol for Action B reinforcement, extinction and contingency degradation. **b**, ChR2-dependent increase in Action B. **c**, Quantification of (**b**). ChR2-YFP and YFP groups differed across time and groups (Two-way ANOVA. F(3,63) = 38.67, p < 0.0001). Tukey’s post hoc comparisons. **d**, Actual stimulation frequency for single action learning. Significant increases across time per group (1-way ANOVA. Action A: F(1.34, 18.8) = 112, p < 0.0001. Action B: F(1.18, 14.2) = 46.5, p < 0.0001). Two-sided Dunnett multiple comparisons test. **e**, Percent target detection resulting in trigger. Tukey plot (median/25^th^-75^th^ percentile box/min-max fences). **f**, Extinction and re-acquisition of Action B showing mean (black) individual curves (light grey lines). **g**, Quantification of (**f**). ChR2-YFP and YFP groups differed across time and groups (Two-way ANOVA. F(2,42) = 32.23, p < 0.0001). ChR2-YFP and YFP animals decreased Action B frequency by the end of extinction, but only ChR2-YFP animals significantly increased Action B frequency by the end of re-acquisition period. 2-sided Tukey’s post hoc multiple comparisons test. **h**, Quantification of raw frequency changes across learning stage and target similarity percentile groups in ChR2-YFP animals. The p value results of two-way mixed effect model analysis is shown in the table. **i**, Quantification of contingency degradation results. Actin B and Laser Trigger frequencies differ across time and groups (Two-way ANOVA. F(2,48) = 26.19, p < 0.0001). 2-sided Tukey’s post hoc multiple comparisons test. **j**, Evolution of pooled action repertoire (n = 341 actions from 10 YFP animals) across action learning from a naïve state. A black parallelogram was set at baseline normalized frequency = 1 to help visualization. **k**, Cross-sectional view of action repertoire frequencies at Early and Late stages. **l**, Same as (**h**) but for YFP animals. See Supplementary Information for statistical/sample details. Source Data

Source Data Extended Data Fig. 9

**Extended Data Fig. 10:**
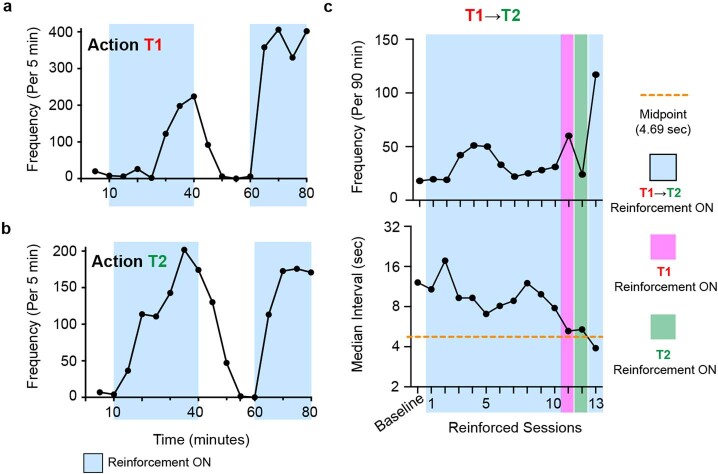
Two-action learning analyses supplementing Fig. 4. **a-c**, Individual ChR2-YFP animal that failed to reach criterion frequency by session 10 and had the largest T1→T2 median interval of all animals at session 10 was tested for the ability to be reinforced for single actions T1 (**a**) and T2 (**b**) in order. Upon verifying that the individual could learn T1 and T2 separately in a ChR2-YFP manner (**a, b**), the animal was subjected again to T1→T2 reinforcement (**c**). **c**, Increased T1→T2 performance (top) coincided with decreased T1→T2 median interval (bottom) below the sigmoidal midpoint (orange dashed lines) fitted in Fig. 4l. Source Data

Source Data Extended Data Fig. 10

**Extended Data Fig. 11:**
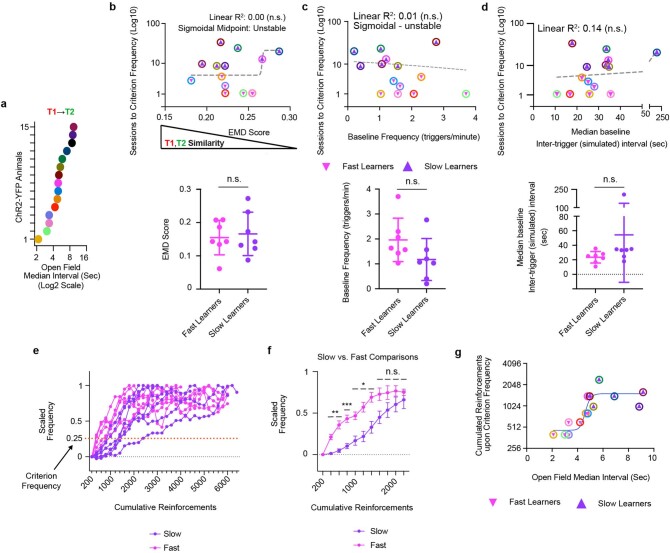
Two-action learning analyses supplementing Fig. 4. **a**, Open field inter-action intervals of T1/T2 pairs chosen. Same colour codes in (**b-d, g**). **b-d**, Top panels: Action similarities between T1 and T2 (**b**) baseline T1 → T2 frequencies (simulated triggers per minute) (**c**) and median baseline inter-simulated trigger intervals (**d**) do not account for variation in sessions to criterion frequency. (F-tests: F(1, 12) = 0.04(**b**), 1.35(**c**), 1.91(**d**): p = 0.85(**b**), p = 0.27(**c**), p = 0.19(**d**). Grey dashed line – model fit (sigmoidal in (**b**) and linear in (**c,d**). Bottom panels: No significant difference between groups in any independent variables shown in the top panels (p = 0.73 (**b**), p = 0.11 (**c**), p = 0.24 (**d**)). Mean +/− s.t.d. 2-tailed, unpaired t-test assuming equal variance. **e**, Individual learning curves scaled to initial session frequency (value of 0) and maximum frequency (value of 1). **f**, Slow learners still show significantly slower learning after matching for number of reinforcements. Plot shows Mean/S.E.M. Significant interaction between cumulative reinforcement bins and groups (2-way ANOVA. F(10, 120) = 5.35) p < 0.0001). 2-tailed Sidak’s multiple comparisons (left to right comparisons on graph: p = 0.0004, 0.002, 0.0005, 0.016, 0.016, 0.03, 0.24, 0.72, 0.86,1). **g**, Sigmoidal relationship between open field T1→T2 interval and cumulated reinforcements upon criterion scaled frequency. *n* = 14 ChR2-YFP mice (biological replicates) (**b-g**). Asterisks: *** p < 0.001. ** p < 0.01. * p < 0.05. n.s. not significant. See Supplementary Information for statistical/sample details. Source Data

Source Data Extended Data Fig. 11

**Extended Data Fig. 12:**
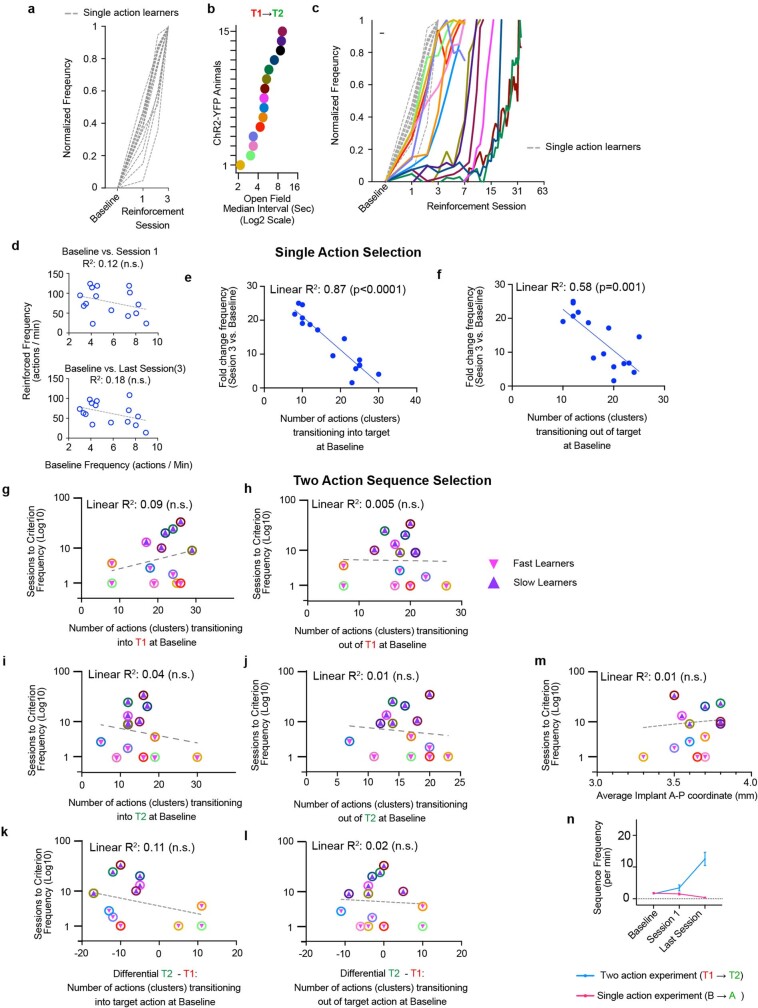
Two-action learning analyses supplementing Fig. 4. **a-c**, Emergence of distinct type of learning curves specifically amongst action sequence learners. **a**, Learning curves of single action learners. Normalized frequency indicates that frequencies were normalized to the highest frequency achieved in a session for each ChR2-YFP animal. **b**, Open field inter-action intervals of T1/T2 pairs chosen in action sequence learning. Same colour codes in (**c, g-m**). c, Overlaying learning curves of single action learners (grey, dashed lines) with action sequence learners (colour coded as in (**b**)). **d-f**, Linear relationships assessed in single action learners. **d**, No significant linear relationship between baseline action frequency and reinforced frequencies in Session 1 (top) and 3 (bottom). **e-f**, The number of action (clusters) transitioning into (**e**) and out of (**f**) target at baseline is negatively related to the fold change in frequency from baseline to last session. F-test (F(1, 13) = 83.31(**e**), 17.88(**f**); p = 5*10^−7^ (**e**), 1.0*10^−3^ (**f**). **g-l**, Action-to-Target (**g, i**) and Target-to-Action (**h,j**) and differential T2-T1 (**k,l**) transitioning parameters are not related to sessions to criterion frequency parameter. F-test: F(1, 12) = 1.11(**g**), 0.0053(**h**), 0.037(**i**), 0.014(**j**), 1.49(**k**), 0.048(**l**); p = 0.31(**g**), 0.94(**h**), 0.51(**i**), 0.69(**j**), 0.24(**k**), 0.83(**l**). Some data points overlap. **m**, No significant linear relationship between implant location and sessions to criterion. F-test: F(1, 12) = 0.11, p = 0.75). Some data points overlap (**g-m**). **n**, Lack of sequence reinforcement in single action reinforcement. Learning curves differ across time and group (2-way ANOVA – F(2, 54) = 143.3, p < 0.0001). Plot indicates mean +/− SEM. *n* = 15 and 14 ChR2-YFP animals (biological replicates) in single action reinforcement and action sequence reinforcement (past criterion frequency) experiments, respectively. n.s. – not significant. Source Data

Source Data Extended Data Fig. 12

**Extended Data Fig. 13:**
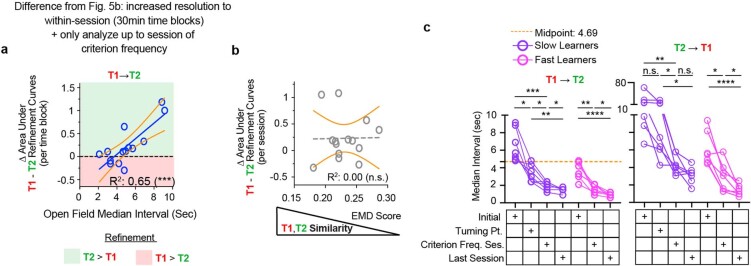
Two-action learning analyses supplementing Fig. 5. **a**, Linear relationship between initial T1→T2 interval and differential T1-T2 refinement, despite accounting for within-session changes and excluding data upon reaching rising phase of learning. Non-zero slope significance: p < 0.001. **b**, Action similarities between T1 and T2 do not account for variation in differential T1-T2 refinement. *n* = 14 ChR2-YFP mice (biological replicates). Black dashed line - linear fit. Grey dashed line - sigmoidal fit. **c**, Full plots of T1→T2 and T2→T1 median interval changes across learning stages. Repeated-measures 2-way ANOVA. time-specific difference (Slow Learners – F(2.184, 26.20) = 54.21, p = 5.3*10^−10^; Fast Learners – F(1.700, 20.40) = 92.12, p = 6.3*10^−9^). Post-hoc 2-tailed Tukey’s multiple comparisons tests. **** p < 0.0001. *** p < 0.001. ** p < 0.01. * p < 0.05. n.s. – not significant. See Supplementary Information for statistical/sample details. Source Data

Source Data Extended Data Fig. 13

**Extended Data Fig. 14:**
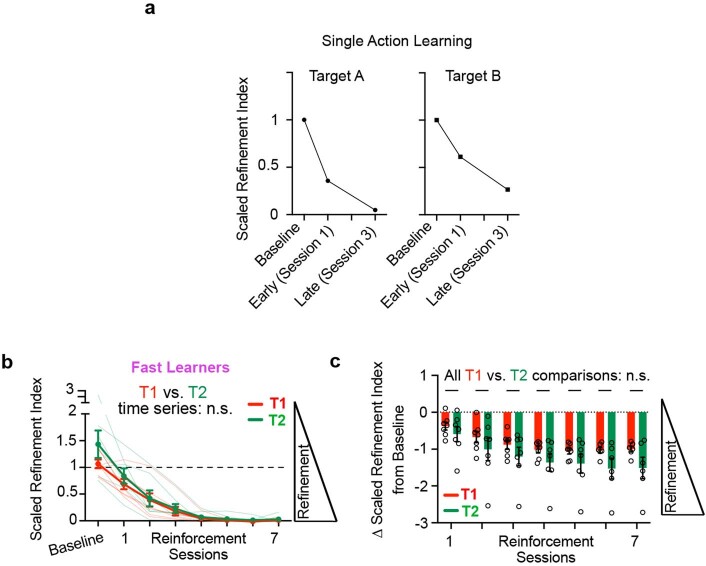
Refinement of T2 and T1 in ChR2-YFP animals. **a**, Patterns of scaled refinement index changes in Single Action Learners, extracted from normalized curves from Figs. 1k and 2e at EMD value of 0.103 (equivalent to 17th percentile - similar to the median EMD of target-related actions in sequence learning refinement analyses. **b-c**, Fast Learners (*n* = 7 mice (biological replicates)) refine T1 and T2 at similar rate. Repeated measures, mixed effects model. No significant difference between time and scaled refinement indices (raw: F(7, 80) = 0.4776, p = 0.85 (**b**); Baseline-subtracted: F(6, 68) = 0.3351, p = 0.92 (**c**)). Post-hoc Šidák test (**c**). * p < 0.05. n.s. – not significant. All summary plot elements indicate mean +/− S.E.M. See Supplementary Information for statistical/sample details. Source Data

Source Data Extended Data Fig. 14

## Data Availability

Source data are available from the corresponding author on reasonable request, and are available at Zenodo (10.5281/zenodo.10146089). Source data are provided with this paper.

## Code availability

MATLAB (MathWorks) codes used for data analysis are available from the corresponding author.
