# Author Correction: Dynamic behaviour restructuring mediates dopamine-dependent credit assignment

**DOI:** 10.1038/s41586-024-08143-z

**Published:** 2024-10-09

**Authors:** Jonathan C. Y. Tang, Vitor Paixao, Filipe Carvalho, Artur Silva, Andreas Klaus, Joaquim Alves da Silva, Rui M. Costa

**Affiliations:** 1 Department of Neuroscience, Zuckerman Mind Brain Behavior Institute, Columbia University, New York, NY USA; 2 Seattle Children’s Research Institute, Center for Integrative Brain Research, Seattle, WA USA; 3 Department of Pediatrics, University of Washington School of Medicine, Seattle, WA USA; 4 Champalimaud Neuroscience Programme, Champalimaud Research, Champalimaud Foundation, Lisbon, Portugal; 5 Kinetikos, Coimbra, Portugal; 6 Open Ephys Production Site, Lisbon, Portugal; 7 Champalimaud Experimental Clinical Research Programme, Champalimaud Research, Champalimaud Foundation, Lisbon, Portugal; 8 NOVA Medical School, Universidade NOVA de Lisboa, Lisbon, Portugal; 9 Aligning Science Across Parkinson’s Collaborative Research Network, Chevy Chase, MD USA; 10 Allen Institute, Seattle, WA USA

**Keywords:** Operant learning, Reward

Correction to: *Nature* 10.1038/s41586-023-06941-5 Published online 13 December 2023

In the version of the article originally published, there was an error in the “Single-action sequence reinforcement” section of Methods regarding the duration of sessions 2 and 3 of single-action reinforcement: “A total of 90 min of closed-loop reinforcement...” should have said 60 min. In addition, the following sentence was missing at the end of the Methods section: “All materials, methods, code and software used are available at Zenodo (https://zenodo.org/records/13249956)”. The errors have been corrected in the HTML and PDF versions of the article.

