# Supplementary material for: Dynamic behaviour restructuring mediates dopamine-dependent credit assignment
Source: Nature. 2023 Dec 13;626(7999):583–92. doi: 10.1038/s41586-023-06941-5 (PMC10866702; doi:10.1038/s41586-023-06941-5)
Supplement: Supplementary file 2 — Reporting Summary [file 41586_2023_6941_MOESM2_ESM.pdf]

nature research

Corresponding author(s): Costa, RM

Last updated by author(s): Nov 16, 2023

Reporting Summary

Nature Research wishes to improve the reproducibility of the work that we publish. This form provides structure for consistency and transparency in reporting. For further information on Nature Research policies, see our Editorial Policies and the Editorial Policy Checklist.

Statistics

For all statistical analyses, confirm that the following items are present in the figure legend, table legend, main text, or Methods section.

n/a	Confirmed	
☐	☒	The exact sample size (*n*) for each experimental group/condition, given as a discrete number and unit of measurement
☐	☒	A statement on whether measurements were taken from distinct samples or whether the same sample was measured repeatedly
☐	☒	The statistical test(s) used AND whether they are one- or two-sided<br>*Only common tests should be described solely by name; describe more complex techniques in the Methods section.*
☐	☒	A description of all covariates tested
☐	☒	A description of any assumptions or corrections, such as tests of normality and adjustment for multiple comparisons
☐	☒	A full description of the statistical parameters including central tendency (e.g. means) or other basic estimates (e.g. regression coefficient) AND variation (e.g. standard deviation) or associated estimates of uncertainty (e.g. confidence intervals)
☐	☒	For null hypothesis testing, the test statistic (e.g. *F*, *t*, *r*) with confidence intervals, effect sizes, degrees of freedom and *P* value noted<br>*Give P values as exact values whenever suitable.*
☒	☐	For Bayesian analysis, information on the choice of priors and Markov chain Monte Carlo settings
☒	☐	For hierarchical and complex designs, identification of the appropriate level for tests and full reporting of outcomes
☐	☒	Estimates of effect sizes (e.g. Cohen's *d*, Pearson's *r*), indicating how they were calculated

Our web collection on statistics for biologists contains articles on many of the points above.

Software and code

Policy information about availability of computer code

Data collection	This is provided in the methods sections. Bonsai v2.3.1 was used for data collection across all modalities.
Data analysis	This is provided in the methods sections. Data analysis and statistical tests were done using Prism Versions 7, 9, 10(GraphPad), Matlab R2022a latest update (MathWorks)., R 2022.07.1+554. Microsoft Excel 16.78.3. (We have begun depositing data on Zenodo (DOI 10.5281/zenodo.10146089) and plan on submitting codes onto GitHub.

For manuscripts utilizing custom algorithms or software that are central to the research but not yet described in published literature, software must be made available to editors and reviewers. We strongly encourage code deposition in a community repository (e.g. GitHub). See the Nature Research guidelines for submitting code & software for further information.

Data

Policy information about availability of data

All manuscripts must include a data availability statement. This statement should provide the following information, where applicable:

- Accession codes, unique identifiers, or web links for publicly available datasets
- A list of figures that have associated raw data
- A description of any restrictions on data availability

We have deposited data relevant to this manuscript on Zenodo (DOI 10.5281/zenodo.10146089). All data that support the findings of this study are available from the corresponding author upon reasonable request

Field-specific reporting

Please select the one below that is the best fit for your research. If you are not sure, read the appropriate sections before making your selection.

☒ Life sciences ☐ Behavioural & social sciences ☐ Ecological, evolutionary & environmental sciences

For a reference copy of the document with all sections, see nature.com/documents/nr-reporting-summary-flat.pdf

Life sciences study design

All studies must disclose on these points even when the disclosure is negative.

Sample size	For sample size, we applied a power of 0.8, significance of p<0.05, and standard variation of 20% of the mean. We determined sample sizes of 4-8 mice per group for different mean-based tests (matched pairs, 2 groups). The experimenter was not blinded of the experimental groups. Optogenetic manipulations were performed automatically via a computer algorithm and not manually by the experimenter.
Data exclusions	For two action sequence learning, significance testing was performed on 14 of 15 ChR2-YFP animals that reached criterion frequency (ChR2-YFP Criterion). The criterion is set such that it is > 20 % above the highest baseline-subtracted frequency value seen at open field condition. The lone animal that did not reach criterion frequency was removed because the T1-->T2 median interval was still very high after session 10, raising doubt as to whether it was capable of learning the reward rule. Thus, this animal was removed from protocol and subjected to single action reinforcement protocol to assess its ability to learn T1 and subsequently T2. Next, the animal was again subjected to T1-->T2 reinforcement protocol . These results indicate that this animal was capable of action learning for both T1 and T2 separately, and also for T1-->T2 sequence after learning of each individual action (Supplementary Figure 10a-c). Thus, it is likely that the animal would have learned the reward rule eventually with more sessions. Removal of the animal did not change the conclusions of the paper. This follow-up results with this animal actually affirms the importance of initial T1-->T2 median interval on influencing learning of a two action reward rule.
Replication	Replication of results were ensured by experimenting on individual mice (>6) per experiment, by performing the same protocol on different mice on different days and assessing consistency of trends seen across mice via statistical approaches. Individual mouse serve as biological replicate. Attempts at replication for the results reported here were successful - the notable exception was a single ChR2-YFP animal that did not reach criterion frequency during two action sequence learning (Extended Data Figure 10). We determined that the lack of learning for this individual was related to our discovery that initial T1-->T2 median interval can impact sequence learning and actually support our conclusions (as we validated this individual's eventual ability to learn with an easier step-wise conditioning (Extended Data Figure. 10). Multiple mouse breeding pairs were used for the experiments to ensure replicability.
Randomization	No formal method of randomization was used; littermates were equally divided among the groups being compared.
Blinding	The experimenter was not blinded of the experimental groups. Optogenetic manipulations were performed automatically via a computer algorithm and not manually by the experimenter. Closed loop reinforcement is difficult to blind because as soon as animals begin to show reinforcement patterns the difference between control and experimental conditions become obvious to the experimenter. It also speaks to the robust ChR2-dependent phenotypes that we are reporting in this study.

Reporting for specific materials, systems and methods

We require information from authors about some types of materials, experimental systems and methods used in many studies. Here, indicate whether each material, system or method listed is relevant to your study. If you are not sure if a list item applies to your research, read the appropriate section before selecting a response.

Materials & experimental systems

n/a	Involved in the study	
☐	☒	Antibodies
☒	☐	Eukaryotic cell lines
☒	☐	Palaeontology and archaeology
☐	☒	Animals and other organisms
☒	☐	Human research participants
☒	☐	Clinical data
☒	☐	Dual use research of concern

Methods

n/a	Involved in the study	
☒	☐	ChIP-seq
☒	☐	Flow cytometry
☒	☐	MRI-based neuroimaging

Antibodies

Antibodies used	Rabbit anti-GFP 488 conjugate (1:1000; Molecular Probes A21311; validated on transfected YFP protein in mammalian HEK-293E lysates by Western Blot: https://www.thermofisher.com/antibody/product/GFP-Antibody-Polyclonal/A-21311). Mouse Anti-TH (1:5000; Immunostar Th 22941; validated on rat midbrain and specified for mouse TH analysis: https://www.immunostar.com/product/tyrosine-hydroxylase-antibody/) with Goat Anti-Mouse - IgG (H+L) Highly cross-adsorbed secondary antibody - Alexa Fluor647 (1:1000; ThermoFisher, A-21236; validated on various mammalian cellular systems: https://www.thermofisher.com/antibody/product/Goat-anti-Mouse-IgG-H-L-Highly-Cross-Adsorbed-Secondary-Antibody-Polyclonal/A-21236)

Validation	Validation is provided in the data sheet of the manufacturer. These reagents have been validated in our system in this study and in our lab in past studies.

Animals and other organisms

Policy information about studies involving animals; ARRIVE guidelines recommended for reporting animal research

Laboratory animals	3-6 month old Mus musculus, C57/Bl6 DAT-Cre male mice. Animals were housed in regular light:dark cycle between 8am:8pm light and 8pm:8am dark. Ambient temperature and humidity were allowed.
Wild animals	No wild animals were used.
Field-collected samples	No field-collected samples were used
Ethics oversight	All experiments were approved by the Portuguese DGAV and Champalimaud Centre for the Unknown Ethical Committee and performed in accordance with European guidelines. Also, the study was performed in accordance to National Institutes of Health (NIH) guidelines and approved by the Institutional Animal Care and Use Committee of Columbia University

Note that full information on the approval of the study protocol must also be provided in the manuscript.
