# Supplementary material for: Dynamic behaviour restructuring mediates dopamine-dependent credit assignment
Source: Nature. 2023 Dec 13;626(7999):583–92. doi: 10.1038/s41586-023-06941-5 (PMC10866702; doi:10.1038/s41586-023-06941-5)
Supplement: Supplementary file 3 — Notes of relevance to the Article. [file 41586_2023_6941_MOESM3_ESM.pdf]

Supplementary Notes

Hypervolume analysis in single action learning

As t-SNE do not include higher dimensional information, we analyzed overlap of action cluster hypervolumes in higher dimensional space (Methods). The range of maximum proportion of an action cluster hypervolume overlapping with target action hypervolume per animal is very low, on the order of 1*10^-5 to 1*10^-30 (Value of 0 is no overlap. Value of 1 indicate perfect overlap between hypervolumes). The percentage of transiently increased actions per animal remain unchanged even after accounting for overlapping action clusters (Wilcoxon 2-tailed test: $p = 0.25$, $n= 15$ ChR2-animals) (Extended Data Fig. 8c). Thus, transiently increased dynamics remain the predominant fate of initially reinforced actions.

Different responses of animals upon naïve reinforcement and target action changes

Whereas naïve animals responded to initial reinforcements for target action A by significantly increasing action A performance relative to the non-target action B (Fig. 2g,i,left graph), animals with a history of reinforcement on action A animals responded to initial reinforcements of action B by increasing non-target action A performance (Fig. 2g,i,right graph). This trend reverses later such that target action B becomes significantly increased over the non-target action A (Fig. 2g,i,right graph). YFP control animals showed no such trends (Fig. 2h,j). Thus, DA reinforcement does not simply reinforce the recently performed, temporally contiguous action, but trigger previously credited actions in the face of a new action-reward contingency that is not yet learned. This suggest again that animals learned the contingency between action performance and DA release.
